# Reptile Search Algorithm Considering Different Flight Heights to Solve Engineering Optimization Design Problems

**DOI:** 10.3390/biomimetics8030305

**Published:** 2023-07-11

**Authors:** Liguo Yao, Guanghui Li, Panliang Yuan, Jun Yang, Dongbin Tian, Taihua Zhang

**Affiliations:** 1 School of Mechanical and Electrical Engineering, Guizhou Normal University, Guiyang 550025, China; lgyao@gznu.edu.cn (L.Y.); ghli@gznu.edu.cn (G.L.); yuanpanl2020@163.com (P.Y.); juny@gznu.edu.cn (J.Y.); 202201001@gznu.edu.cn (D.T.); 2 Technical Engineering Center of Manufacturing Service and Knowledge Engineering, Guizhou Normal University, Guiyang 550025, China

**Keywords:** reptile search algorithm, engineering optimization design, northern goshawk optimization, artificial vulture optimization algorithm

## Abstract

The reptile search algorithm is an effective optimization method based on the natural laws of the biological world. By restoring and simulating the hunting process of reptiles, good optimization results can be achieved. However, due to the limitations of natural laws, it is easy to fall into local optima during the exploration phase. Inspired by the different search fields of biological organisms with varying flight heights, this paper proposes a reptile search algorithm considering different flight heights. In the exploration phase, introducing the different flight altitude abilities of two animals, the northern goshawk and the African vulture, enables reptiles to have better search horizons, improve their global search ability, and reduce the probability of falling into local optima during the exploration phase. A novel dynamic factor (*DF*) is proposed in the exploitation phase to improve the algorithm’s convergence speed and optimization accuracy. To verify the effectiveness of the proposed algorithm, the test results were compared with ten state-of-the-art (SOTA) algorithms on thirty-three famous test functions. The experimental results show that the proposed algorithm has good performance. In addition, the proposed algorithm and ten SOTA algorithms were applied to three micromachine practical engineering problems, and the experimental results show that the proposed algorithm has good problem-solving ability.

## 1. Introduction

With the deeper exploration of natural laws by humans, more and more practical problems have emerged in fields such as control [1,2], manufacturing [3,4], economics [5,6], and physics [7]. Most of these problems have characteristics such as a large scale, multiple constraints, and discontinuity [8]. Traditional algorithms often optimize the objective function results through the gradient of the objective function, a deterministic search method that makes it difficult for people to use existing traditional methods to solve such problems.

Basically, the characteristic of most heuristic algorithms is random search, and through this characteristic, higher global optimal possibilities are obtained [9]. Due to their independence from utilizing function gradients, heuristic algorithms do not require the objective function to have continuously differentiable conditions, providing optimization possibilities for some objective functions that cannot be optimized through gradient descent. Heuristic algorithms can be roughly divided into three categories based on the different ideas of imitation: simulating biological habits [10,11], cognitive thinking [12,13], and physical phenomena [14,15]. Among these, due to the abundance of natural organisms, heuristic algorithms that simulate bodily patterns are primarily used, such as the genetic algorithm (GA) [16], particle swarm optimization (PSO) [17], ant colony optimization (ACO) [18], Grey wolf optimizer (GWO) [19], etc. However, no free lunch globally exists, and no single algorithm is suitable for solving all optimization problems [20]. In recent years, in pursuit of the effectiveness of heuristic algorithms, many improved algorithms have emerged, mainly consisting of strategy-based improvement and algorithm combinations. In recent years, our research team has been committed to obtaining better-performing heuristic algorithms through algorithmic combinations, such as the beetle antenna strategy based on grey wolf optimization [21], grey wolf optimization based on the Aquila exploration method (AGWO) [22], hybrid golden jackal optimization and the golden sine algorithm [23], enhanced snake optimization [24], etc.

The reptile search algorithm (RSA) is a novel intelligent optimization algorithm based on crocodile hunting behavior that was proposed by Laith et al. in 2022 [25]. The RSA has the characteristics of fewer parameter adjustments, strong optimization stability, and easy implementation, achieving excellent results in optimization problems. Ervural and Hakli proposed a binary RSA to extend the RSA to binary optimization issues [26]. Emam et al. proposed an enhanced reptile search algorithm for global optimization. They selected the optimal thresholding values for multilevel image segmentation [27]. Xiong et al. proposed a dual-scale deep learning model based on ELM-BiLSTM and improved the reptile search algorithm for wind power prediction [28]. Elkholy et al. proposed an AI-embedded FPGA-based real-time intelligent energy management system using a multi-objective reptile search algorithm and a gorilla troops optimizer [29].

However, due to the physiological limitations of any animal, there are corresponding drawbacks to algorithms that simulate biological habits. This also leads to the RSA, like other algorithms that simulate physical patterns, having a slow convergence speed, low optimization accuracy, and being prone to falling into local optima. This article aims to solve this problem by studying the natural patterns of organisms inspired by natural laws. Crocodiles have good hunting ability as land animals but need a better observation field due to height constraints. Therefore, in the search section, the performance could be better (in line with the RSA’s slow convergence speed, low optimization accuracy, and quick fall into local optima). Inspired by the different flight heights and search horizons of natural organisms, this article introduces the African vulture optimization algorithm (AVOA) [30] and northern goshawk optimization (NGO) [31], utilizing the high-altitude advantages of birds to explore accordingly. Considering the sizeable spatial range, the northern goshawk algorithm is used in the high-altitude field, and African vulture optimization is used in the mid- to high-altitude range. In the exploration phase, the hunting advantages of crocodiles are utilized. On this basis, a reptile search algorithm considering different flight heights (FRSA) is proposed.

To verify the effectiveness of the FRSA, a comparison was made with ten SOTA algorithms on two function sets (thirty-three functions) and three engineering design optimization problems, demonstrating significant improvements in both the algorithm’s performance and its practical problem-solving capabilities. The highlights and contributions of this paper are summarized as follows: (1) The reptile search algorithm considering different flight heights is proposed. (2) Wilcoxon rank sum and Friedman tests are used to analyze the statistical data. (3) The FRSA is applied to solve three constrained optimization problems in mechanical fields and compared with ten SOTA algorithms.

The rest of this article is arranged as follows: Section 2 reviews the RSA, and Section 3 provides a detailed introduction to the FRSA, including all the processes of exploration and exploitation. Section 4 describes and analyzes the results of the FRSA and other comparative algorithms on the two sets of functions. Section 5 represents the FRSA’s performance on three practical engineering design issues. Finally, Section 6 provides a summary and the outlook of the entire article.

## 2. RSA

The RSA is a novel, naturally inspired meta-heuristic optimizer. It simulates the hunting behavior of crocodiles to optimize problems. Crocodiles’ hunting behavior is divided into two phases: implement encirclement (exploration) and hunting (exploitation). The implementation of hunting is achieved through high walking or belly walking, and hunting is achieved through hunting coordination or hunting cooperation.

In each optimization process, the first step is to generate an initial population. In the RSA, the initial population of crocodiles is randomly generated, as described in Equation (1), and the rules for randomly generating populations are shown in Equation (2).

(1) $$P={\left[\begin{array}{c} p_{1}\\ p_{2}\\ \vdots \\ p_{m} \end{array}\right]}_{m\times n}={\left[\begin{array}{cccc} p_{1,1} & p_{1,2} & \ldots & p_{1,n}\\ p_{2,1} & p_{2,2} & \ldots & p_{2,n}\\ \vdots & \vdots & \vdots & \vdots \\ p_{m,1} & p_{m,2} & \ldots & p_{m,n} \end{array}\right]}_{m\times n}$$

where P denotes randomly generated initial solutions, and $p_{m,n}$ represents the position of the *m*-th solution in the *n*-th dimension. m denotes the number of candidate solutions, and *n* denotes the dimension of the given problem.

(2) $$p_{i,j}=rand_{1}\times \left(ub-lb\right)+lb,\;j=1,2,\cdots ,n$$

where $rand_{1}$ denotes a random value between 0 and 1, and lb and ub denote the lower and upper bounds of the given problem, respectively.

The RSA can transition between encirclement (exploration) and hunting (exploitation), and each phase can be divided into two states according to different situations. Therefore, the RSA can be divided into four other parts.

During the exploration phase, there are two states: high-altitude walking and abdominal walking. When t≤T/4, the crocodile population enters a high-altitude walking state, and when T/4<t≤T/2, the crocodile population enters an abdominal walking state. Different conditions during the exploration phase benefit the population by conducting better searches and finding better solutions. The position update rules of the population during the exploration phase are shown in Equation (3).

(3) $$p_{i,j}^{t+1}=\left\{ \begin{array}{cc} {\mathrm{Best}}_{j}^{t}\times \delta_{i,j}^{t}\times 0.1-R_{i,j}^{t}\times rand_{2} & t\leq \frac{T}{4}\\ {\mathrm{Best}}_{j}^{t}\times p_{r_{1},j}\times ES^{t}\times rand_{3} & \frac{T}{4}<t\leq \frac{T}{2}\; \end{array}\right.$$

where ${\mathrm{Best}}_{j}^{t}$ denotes the position of the optimal solution at time t in the *j*-th dimension, T is the maximum number of iterations per experiment, and $rand_{2}$ and $rand_{3}$ denote a random value between 0 and 1. $\delta_{i,j}^{t}$ denotes the hunting operator for the *j*-th dimension of the *i*-th candidate solution, which can be calculated by Equation (4). $R_{i,j}^{t}$ is a scaling function used to reduce the search area, which can be calculated by Equation (5). $r_{1}$ is a random number between 1 and *m*, and $ES^{t}$ is an evolutionary factor, with a randomly decreasing value between 2 and −2, which can be calculated by Equation (6).

(4) $$\delta_{i,j}^{t}={\mathrm{Best}}_{j}^{t}\times d_{i,j}$$

(5) $$R_{i,j}^{t}=\frac{{\mathrm{Best}}_{j}^{t}-p_{r_{2},j}}{{\mathrm{Best}}_{j}^{t}+\theta }$$

(6) $$ES^{t}=2\times rand_{4}\times (1-\frac{t}{T})$$

where θ is a near-zero minimum, which is to prevent cases where the denominator is zero, $rand_{4}$ is an integer between −1 and 1, and $d_{i,j}$ represents the percentage difference between the best solution and the current solution in the *j*-th dimension position, which can be calculated by Equation (7).

(7) $$d_{i,j}=0.1+\frac{p_{i,j}-\frac{1}{n}\sum_{j=1}^{n}p_{i,j}}{{\mathrm{Best}}_{j}^{t}\times (ub_{j}-lb_{j})+\theta }$$

In the exploitation phase, there are two states based on the hunting behavior of crocodiles: hunting coordination and hunting cooperation. Crocodile hunting coordination and cooperation enable them to approach their target prey easily, as their reinforcement effect differs from the surrounding mechanism. Therefore, exploitation search may discover near-optimal solutions after several attempts. When T/2<t≤3T/4, the crocodile population enters a hunting coordination state, when 3T/4<t≤T, the crocodile population enters a hunting cooperative state. Different states during the exploitation phase are beneficial in avoiding optimization from falling into local optima and helping to determine the optimal solution during the exploitation phase. The location update rules of the population during the exploration phase are shown in Equation (8).

(8) $$p_{i,j}^{t+1}=\left\{ \begin{array}{ll} {\mathrm{Best}}_{j}^{t}\times d_{i,j}^{t}\times rand_{5} & \frac{T}{2}<t\leq \frac{3T}{4}\;\\ {\mathrm{Best}}_{j}^{t}-\delta_{i,j}^{t}\times \theta -R_{i,j}^{t}\times rand_{6} & \frac{3T}{4}<t\leq T\; \end{array}\right.$$

where ${\mathrm{Best}}_{j}^{t}$ denotes the position of the optimal solution at time t in the *j*-th dimension, and $rand_{5}$ and $rand_{6}$ denote random values between 0 and 1. $R_{i,j}^{t}$ is a scaling function used to reduce the search area, which can be calculated by Equation (5). θ is a minimal value.

The pseudo-code of the RSA is shown in Algorithm 1.
**Algorithm 1.** Pseudo-code of RSA1.Define *Dim*, *UB*, *LB*, Max_Iter(*T*), Curr_Iter(*t*), *α*, *β*, etc2.Initialize the population randomly $p_{i}(i=1,2,\ldots ,m)$
3.**while** (*t* < *T*) **do**4. Evaluate the fitness of each $p_{i}(i=1,2,\ldots ,m)$
5. Find Best solution6. Update the *ES* using Equation (6).7. **for** (*i* = 1 to m) **do**8.  **for** (*j* = 1 to *n*) **do**9.  Update the *η*, *R*, *P* and values using Equations (4), (5) and (7), respectively.10.  **If** (*t* ≤ *T*/4) **then**11.   Calculate $p_{i,j}^{t+1}$ using Equation (3)12.  **else if** (*t* ≤ 2*T*/4 and *t* > *T*/4) **then**13.   Calculate $p_{i,j}^{t+1}$ using Equation (3)14.  **else if** (*t* ≤ 3*T*/4 and *t* > 2*T*/4) **then**15.   Calculate $p_{i,j}^{t+1}$ using Equation (8)16.  **else**
17.   Calculate $p_{i,j}^{t+1}$ using Equation (8)19.   **end if**
20.  **end for**
21. **end for**
22. t = t+123.**end while**24.Return the best solution.

## 3. Proposed FRSA

As a heuristic algorithm, the RSA has achieved good results in solving optimization problems due to its novel imitation approach. However, due to the limitations of natural biological behavior, this algorithm still has some drawbacks. In the process of individual optimization, multiple complex situations may be encountered, and the steady decrease in evolutionary factors does not conform to the nonlinear optimization law of algorithms when dealing with complex optimization problems. The team collaboration, search scope, and hunting mechanism of the crocodile population are all updated around the current optimal value. The iterative updating process of individuals lacks a mutation mechanism. Suppose the present optimal individual falls into a local optimum. In that case, it is easy for the population to aggregate quickly, resulting in the algorithm being unable to break free from the constraints of the local extremum.

In this section, based on the shortcomings of the RSA, the FRSA is proposed by introducing different search mechanisms (based on the exploration altitude) in the exploration phase of the algorithm and introducing fluctuation factors in the exploration phase.

### 3.1. High-Altitude Search Mechanism (Northern Goshawk Exploration)

The northern goshawk randomly selects prey during the prey identification stage of hunting and quickly attacks it. Due to the random selection of targets in the search space, this stage increases the exploration capability of the NGO algorithm. This stage conducts a global search of the search space to determine the optimal region. At this stage, the behavior of northern goshawks in prey selection and attack is described using Equations (9) and (10).

(9) $$p_{i,j}^{t+1}=\left\{ \begin{array}{l} p_{i,j}^{t}+\left(y_{i,j}^{t}-I\times p_{i,j}\right)\times rand_{7}\;\;F_{y_{i}}<F_{i}\;\\ p_{i,j}^{t}+\left(p_{i,j}-y_{i,j}^{t}\right)\times rand_{8}\;\;\;F_{y_{i}}\geq F_{i}\; \end{array}\right.$$

(10) $$P_{i}^{t+1}=\left\{ \begin{array}{l} P_{i}^{t+1}\;\;\;F_{i}^{new}<F_{i}\;\\ P_{i}^{t}\;\;\;\;\;F_{i}^{new}\geq F_{i}\;\; \end{array}\right.$$

where $y_{i}$ is the prey position of the *i*-th northern hawk, $F_{y_{i}}$ is the objective function value of the prey position of the *i*-th northern hawk, $P_{i}^{t+1}$ is the position of the *i*-th northern hawk, $p_{i,j}^{t+1}$ is the position of the *i*-th northern hawk in the *j*-th dimension at time *t*, $F_{i}^{new}$ is the updated objective function value of the *i*-th northern hawk, *I* is a random integer of 1 or 2.

### 3.2. Low-Altitude Search Mechanism (African Vulture Exploration)

Inspired by the speed at which vultures feed or starve, mathematical modeling is performed using Equation (11), which can be used to simulate the exploration and exploration phases. The satiety rate shows a decreasing trend, and this behavior is simulated using Equation (12).

(11) $$\tau =h\times \left(\sin^{\theta }\left(\frac{\pi }{2}\times \frac{t}{T}\right)+\cos\left(\frac{\pi }{2}\times \frac{t}{T}\right)-1\right)$$

(12) $$\eta =\left(2\times rand_{9}+1\right)\times z\times \left(1-\frac{t}{T}\right)+\tau$$

where η represents the hunger level of vultures, t is the current number of iterations, T is the maximum number of iterations, z denotes a random value between −1 and 1, and h denotes a random value between −2 and 2. When |η|>1, the vultures are in the exploration phase. Based on the living habits of vultures, there are two different search methods in the exploration phase of the African vulture optimization algorithm, as shown in Equation (13).

(13) $$p_{i,j}^{t+1}=\left\{ \begin{array}{l} Bes{t^{t}}_{j}-\left|2\times rand_{10}\times Bes{t^{t}}_{j}-p_{i,j}^{t}\right|\times \eta \;\;\;\;\;\;\;\delta \leq 0.6\\ Bes{t^{t}}_{j}-\eta +\;rand_{11}\;\times \left(\left(ub-lb\right)\times rand+lb\right)\;\;\delta >0.6\; \end{array}\right.$$

### 3.3. Novel Dynamic Factor

**Figure 1:**
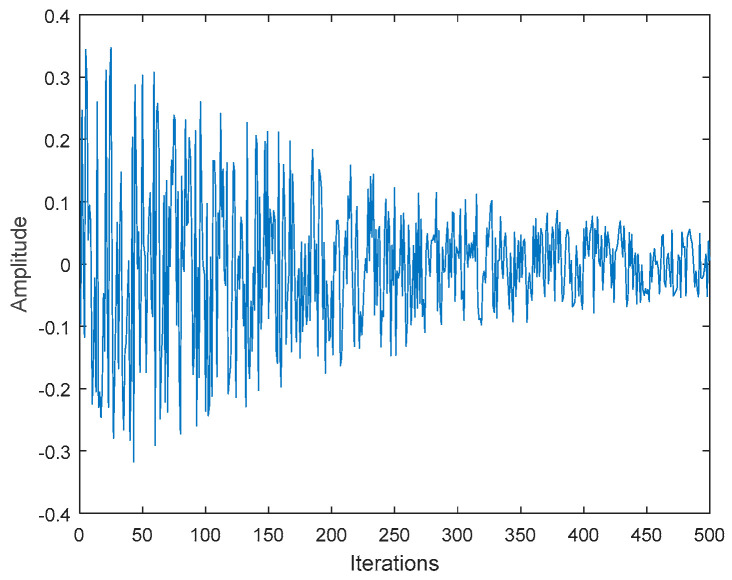
The dynamic factor graph for 500 iterations.

In the exploration phase of the RSA, due to the lack of the random walkability of the algorithm, the convergence speed of the algorithm is slow, and the optimization accuracy is low at this stage. Therefore, this paper proposes a new *DF* on the original basis to add disturbance factors and to improve the random walkability of the algorithm in the exploration stage, enable the population to explore local regions in small steps, reduce the probability of individuals falling into the local extremum under the influence of fluctuations, and improve the optimization accuracy of the algorithm. The new *DF* is calculated by Equation (14). The *DF* graph for 500 iterations is shown in Figure 1.

(14) $$DF=0.4\times (2\times r-1)\times e^{{\left(-t/T\right)}^{2}}$$

where t is the current number of iterations, T is the maximum number of iterations, and r denotes a random value between 0 and 1.

(15) $$p_{i,j}^{t+1}=\left\{ \begin{array}{l} ES\times Bes{t^{t}}_{j}\times d_{i,j}^{t}\times rand_{5}\;\;\;\;\;\;\;\;\;\frac{6T}{10}<t\leq \frac{8T}{10}\;\\ Bes{t^{t}}_{j}-ES\times \delta_{i,j}^{t}\times \theta -R_{i,j}^{t}\times rand_{6}\;\;\;\frac{8T}{10}<t\leq T\;\; \end{array}\right.$$

After adding disturbance factors, the position update rules of the FRSA during the exploration phase are shown in Equation (15).

**Figure 2:**
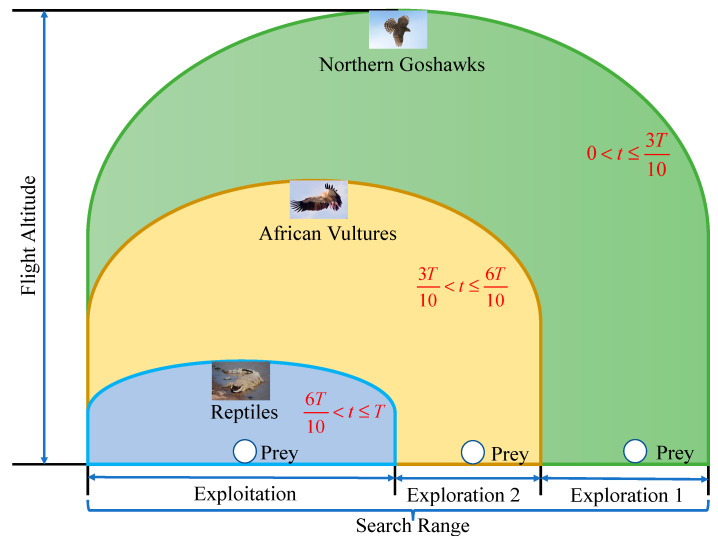
Cooperative hunting mode of FRSA.

**Figure 3:**
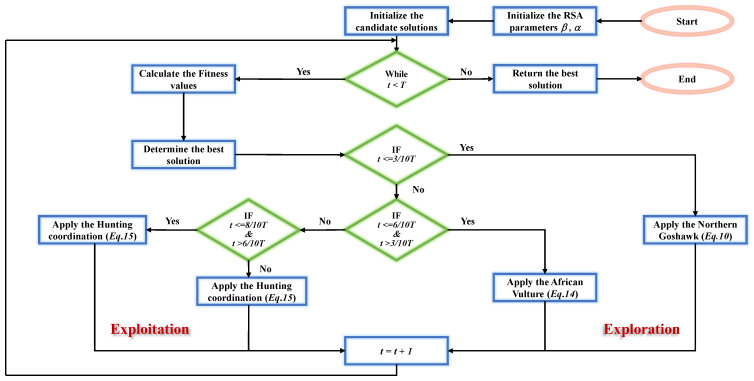
Flowchart of FRSA.

By utilizing the proposed strategy to improve the RSA, the optimization ability and efficiency of RSA can be effectively improved. The cooperative hunting mode of the FRSA is shown in Figure 2. The pseudocode of the FRSA is shown in Algorithm 2. And the flowchart of FRSA is shown in Figure 3.
**Algorithm 2.** Pseudo-code of FRSA1.Define *Dim*, *UB*, *LB*, Max_Iter(*T*), Curr_Iter(*t*), *α*, *β*, etc2.Initialize the population randomly $p_{i}(i=1,2,\ldots ,m)$
3.**while** (*t* < *T*) **do**4. Evaluate the fitness of each $p_{i}(i=1,2,\ldots ,m)$
5. Find Best solution6. Update the *ES* using Equation (6).7. **for** (*i* = 1 to *m*) **do**8.  **for** (*j* = 1 to *n*) **do**9.  Update the *η*, *R*, *P* and values using Equations (4), (5) and (7), respectively.10.  **if** (*t* ≤ 3*T*/10) **then**11.   Calculate $p_{i,j}^{t+1}$ using Equation (10)12.  **else if** (*t* ≤ 6*T*/10 and *t* > 3*T*/10) **then**13.   Calculate $p_{i,j}^{t+1}$ using Equation (14)14.  **else if** (*t* ≤ 8*T*/10 and *t* > 6*T*/10) **then**15.   Calculate $p_{i,j}^{t+1}$ using Equation (15)16.  **else**
17.   Calculate $p_{i,j}^{t+1}$ using Equation (15)18.   **end if**
19.  **end for**
20. **end for**
21. t = t + 122.**end while**23.Return best solution.

### 3.4. Computational Time Complexity of the FRSA

In the process of optimizing practical problems, in addition to pursuing accuracy, time is also an essential element [32]. The time complexity of an algorithm is an important indicator for measuring the algorithm. Therefore, it is necessary to analyze the time complexity of the improved algorithm compared to the original algorithm. The time complexity is mainly reflected in the algorithm’s initialization, fitness evaluation, and update solution.

When there are *N* solutions, the time complexity of the initialization phase is O (N), and the time complexity of the update phase is O (T×N)+O(T×N×D). Therefore, the algorithm complexity of the RSA can be obtained as O (N×(T×D+1)). Compared to the RSA, the time complexity of the FRSA only increases the part of the evolution factor. Assuming the time of the evolution factor is t, the time complexity of the FRSA is O (N×(T×D+1)+t)=O(N×(T×D+1)). From this, the FRSA proposed in this article does not increase the time complexity.

## 4. Analysis of Experiments and Results 

### 4.1. Benchmark Function Sets and Compared Algorithms

This section uses the classic function set and the CEC 2019 set as the benchmark test functions for this article. There are 33 functions, including 7 unimodal, 6 multimodal, and 20 fixed-dimensional multimodal functions. Unimodal functions were used to test the exploration ability of the optimization algorithms due to having only one extreme value. Multimodal functions were used to test the exploration ability of optimization algorithms due to the existence of multiple extreme values. Finally, fixed dimensional parts were used to evaluate the algorithm’s total capacity for exploration and exploration. The details of the classic function set are shown in Table 1. The details of the CEC 2019 set are shown in Table 2.

To better compare the results with other algorithms, this study used ten well-known algorithms as benchmark algorithms, including the GA [16], PSO [17], ACO [18], GWO [19], GJO [33], SO [34], TACPSO [35], AGWO [36], EGWO [36], and the RSA [25]. These benchmark algorithms have achieved excellent results in function optimization and are often used as benchmark comparison algorithms. The details of the parameter settings for the algorithms are shown in Table 3. To be fair, the setting information for these parameters was taken from the original literature that proposed these algorithms.

To fairly compare the results of the benchmark algorithms, all algorithms adopted the following unified parameter settings: the number of independent continuous runs of the algorithm was 30, the number of populations was 50, the number of algorithm iterations was 500, and the comparison indicators included the mean, the standard deviation, the *p*-value, the Wilcoxon rank sum test, and the Friedman test [37,38]. The best results of the test are displayed in bold. This simulation testing environment was carried out on a computer with the following features: Intel(R) Core (TM) i5-9400F CPU @ 2.90 GHz and 16 GB RAM, Windows 10, 64-bit operating system.

### 4.2. Results Comparison and Analysis

To fully validate the robustness and effectiveness of the algorithm for different dimensional problems, this study adopted three dimensions (30, 100, 500) for the non-fixed dimensional functions (unimodal and multimodal functions).

**Figure 4:**
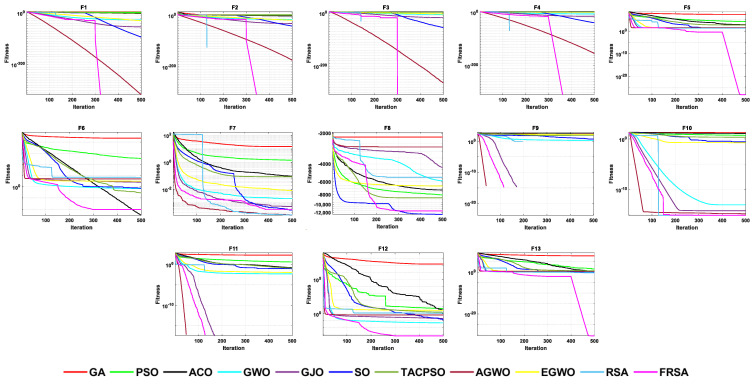
The convergence curves of the 11 algorithms with Dim = 30.

**Figure 5:**
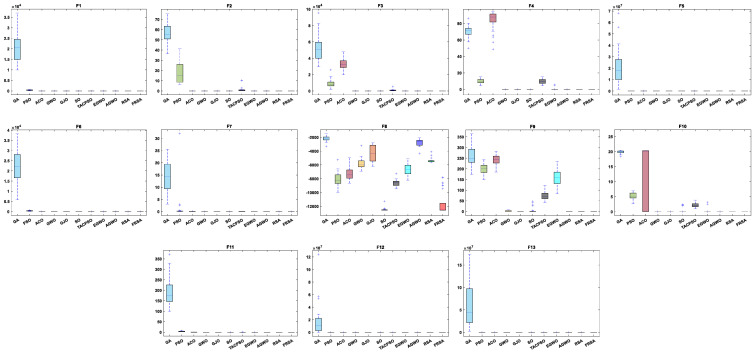
Boxplot analysis of classic functions (F1−F13) with Dim = 30.

Table 4 shows the results of the non-fixed dimensional functions in 30 dimensions, including the mean (Mean), standard deviation (Std), and Friedman test of 11 algorithms. Figure 4 shows the iterative curves of these 11 algorithms for solving 13 non-fixed dimensional functions. Figure 5 is a boxplot of the results obtained by these 11 algorithms after solving 13 functions with non-fixed dimensions. The boxplot results were analyzed from five perspectives: the minimum, lower quartile, median, upper quartile, and maximum. By convergence curves and boxplots, the algorithm can be more intuitively and comprehensively characterized for solving functional problems. Out of 13 non-fixed dimensional functions, the FRSA achieved ten optimal values, with the highest number among all 11 algorithms. The Friedman value shows the overall results obtained by each algorithm in 13 functions. In the Friedman value, the FRSA achieved the mark of 2.2115, ranking first in the Friedman rank, indicating that the FRSA achieved better results than the other algorithms in 30 dimensions.

**Figure 6:**
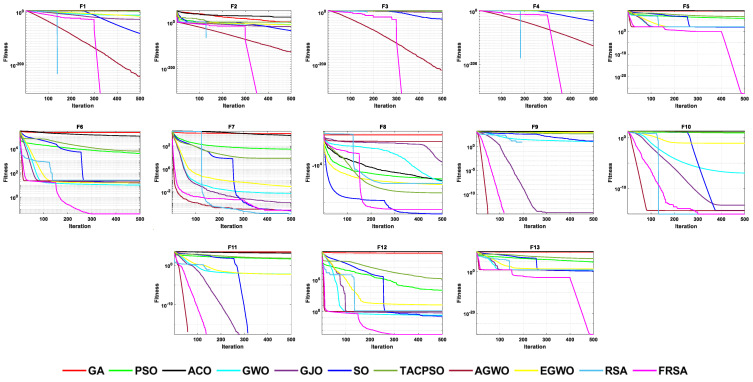
The convergence curves of the 11 algorithms with Dim = 100.

**Figure 7:**
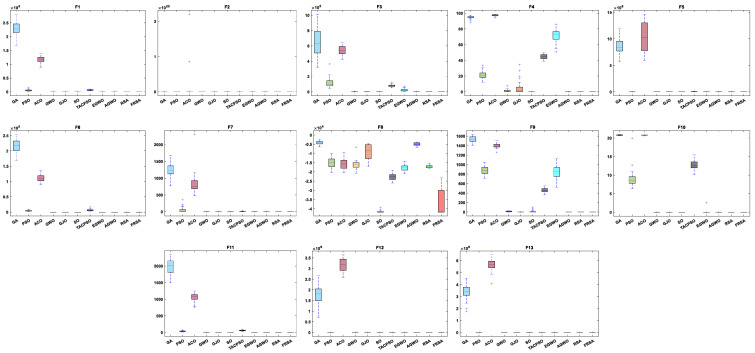
Boxplot analysis of classic functions (F1−F13) with Dim = 100.

Table 5 shows the results of the non-fixed dimensional functions in 100 dimensions, including the Mean, Std, and Friedman test of 11 algorithms. Figure 6 shows the iterative curves of these 11 algorithms for solving 13 non-fixed dimensional functions. Figure 7 is a boxplot of the results obtained by these 11 algorithms after solving 13 functions with non-fixed dimensions. The boxplot results were analyzed from five perspectives: the minimum, lower quartile, median, upper quartile, and maximum. By convergence curves and boxplots, the algorithm can be more intuitively and comprehensively characterized for solving functional problems. Out of the 13 non-fixed dimensional functions, the FRSA achieved 11 optimal values, with the highest number among all 11 algorithms. The Friedman value shows the overall results obtained by each algorithm in the 13 functions. For the Friedman value, the FRSA achieved a mark of 2.0192, ranking first in the Friedman test, and indicating that the FRSA achieved better results than the other algorithms in 100 dimensions.

**Figure 8:**
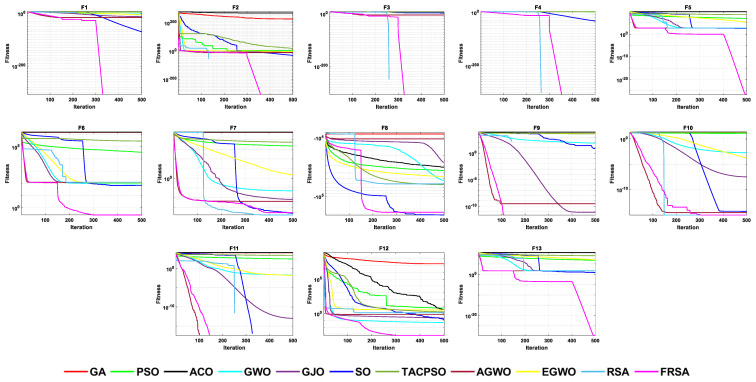
The convergence curves of the 11 algorithms with Dim = 500.

**Figure 9:**
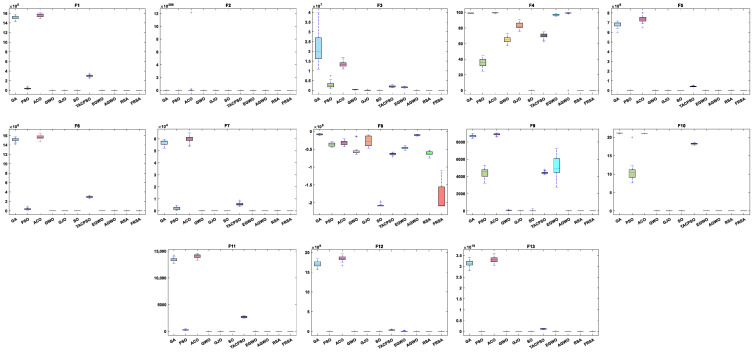
Boxplot analysis of classic functions (F1−F13) with Dim = 500.

Table 6 shows the results of non-fixed dimensional functions at 500 dimensions, including the Mean, Std, and Friedman test of 11 algorithms. Figure 8 shows the iterative curves of these 11 algorithms for solving 13 non-fixed dimensional functions. Figure 9 is a boxplot of the results obtained by these 11 algorithms after solving 13 functions with non-fixed dimensions. The boxplot results were analyzed from five perspectives: the minimum, lower quartile, median, upper quartile, and maximum. By convergence curves and boxplots, the algorithm can be more intuitively and comprehensively characterized for solving functional problems. Out of the 13 non-fixed dimensional functions, the FRSA achieved 11 optimal values, with the highest number among all 11 algorithms. The Friedman value shows the overall results obtained by each algorithm in the 13 functions. For the Friedman value, the FRSA achieved a mark of 1.9615, ranking first in the Friedman test, and indicating that the FRSA achieved better results than the other algorithms in 500 dimensions.

**Figure 10:**
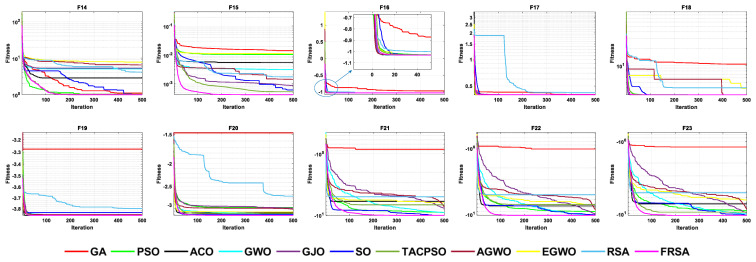
The convergence curves of the 11 algorithms with fixed dimensions.

**Figure 11:**
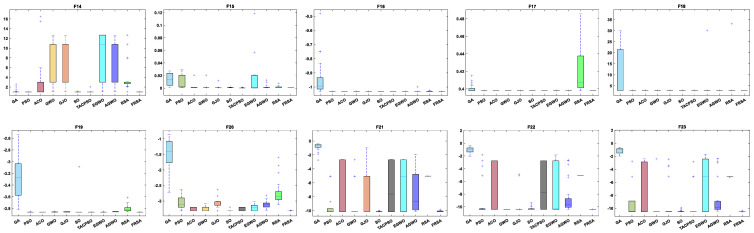
Boxplot analysis of classic functions (F14−F23) with fixed dimensions.

Table 7 shows the results of the fixed dimensional functions, including the Mean, Std, and Friedman test of 11 algorithms. Figure 10 shows the iterative curves of these 11 algorithms for solving 10 fixed dimensional functions. Figure 11 is a boxplot of the results obtained by these 11 algorithms after solving 13 functions with non-fixed dimensions. The boxplot results were analyzed from five perspectives: the minimum, lower quartile, median, upper quartile, and maximum. By convergence curves and boxplots, the algorithm can be more intuitively and comprehensively characterized for solving functional problems. The FRSA achieved 8 optimal values out of the 10 fixed dimensional functions, with the highest number among all 11 algorithms. The Friedman value shows the overall results obtained by each algorithm in the 13 functions. For the Friedman value, the FRSA achieved a mark of 1.9615, ranking first in the Friedman test, and indicating that the FRSA achieved better results than other algorithms in 500 dimensions.

To compare the results of the FRSA with 10 benchmark algorithms more comprehensively, this article introduces another statistical analysis method, the Wilcoxon rank sum test.

As a non-parametric rank sum hypothesis test, the Wilcoxon rank sum test is frequently used in statistical practice for the comparison of measures of location when the underlying distributions are far from normal or not known in advance [39]. The purpose of the Wilcoxon rank sum test is to test whether there is a significant difference between two populations that are identical except for the population mean. In view of this, this article uses the Wilcoxon rank sum test to compare the differences among the results of various algorithms.

For the Wilcoxon rank sum test, the significance level was set to 0.05, and the symbols “+”, “=”, and “-” indicate that the performance of the FRSA was superior, similar, and inferior to the corresponding algorithm, respectively. In Table 8, no underline represents “+”, and “=” and “-” are represented by different underlines: “ ” and “ ”. Thus, it is possible to evaluate the adopted algorithms from multiple perspectives. Table 8 shows the rank sum test results between the FRSA and the ten benchmark algorithms.

**Figure 12:**
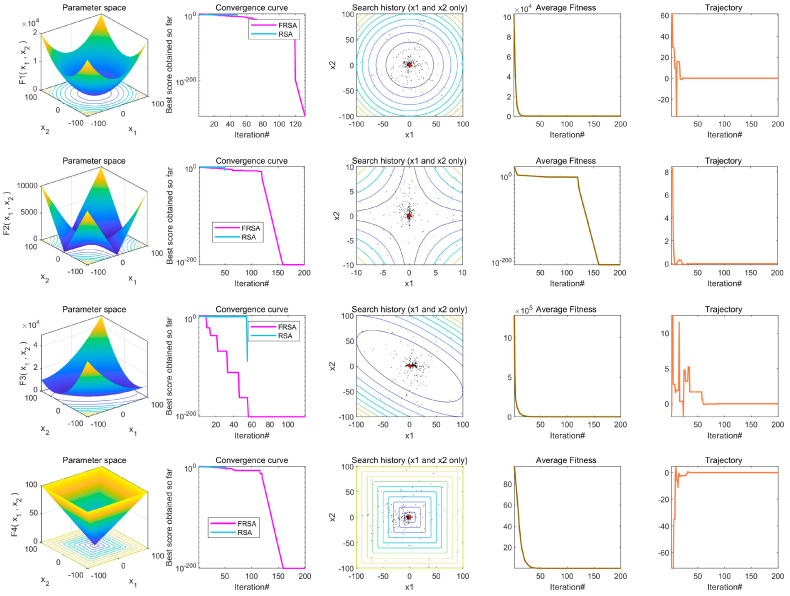

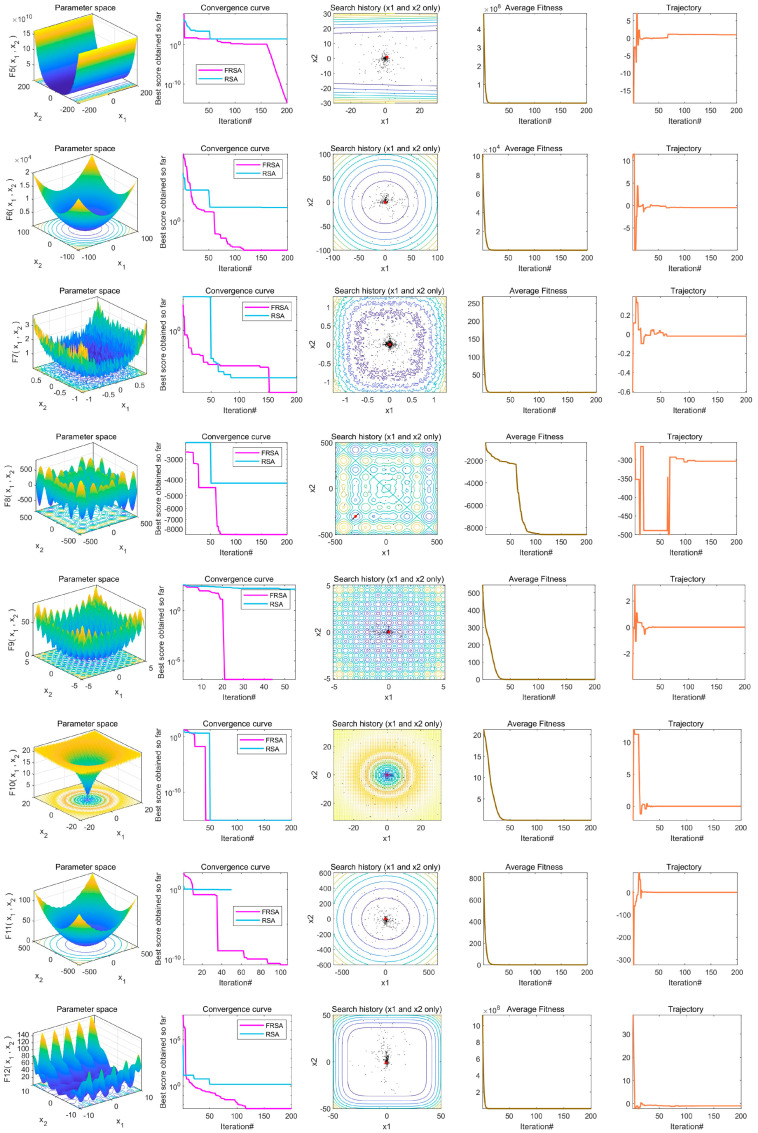

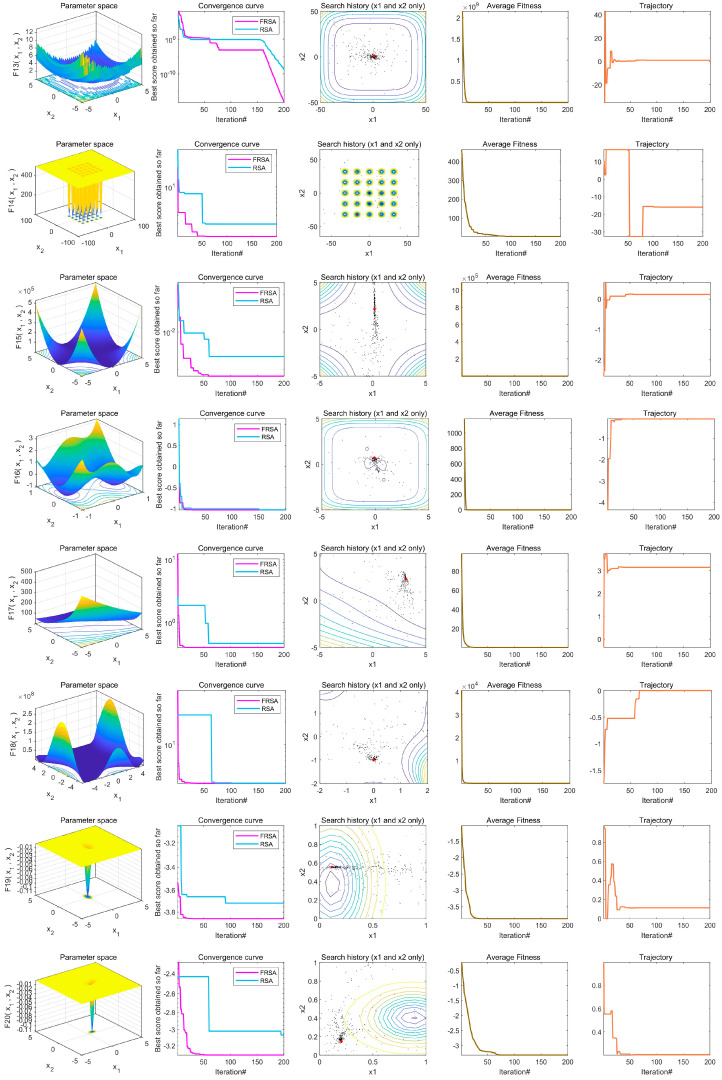

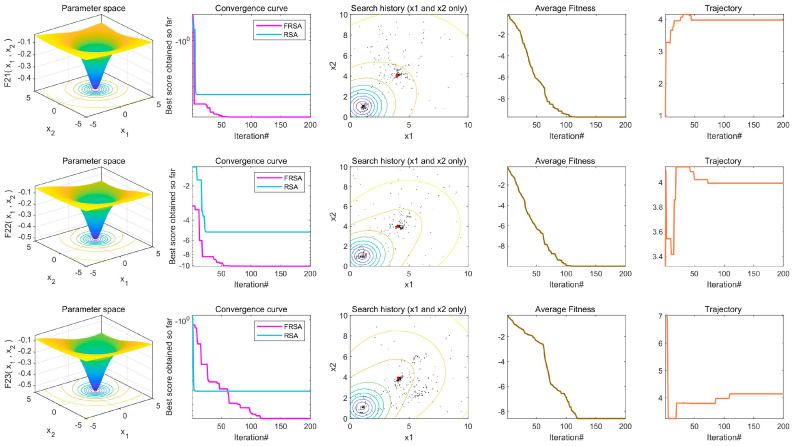
Convergence analysis between RSA and FRSA.

In order to better demonstrate the comparison of the results between the RSA and the FRSA, this study added a comparative analysis of the convergence of the two algorithms, as shown in Figure 12. There are five columns in Figure 12, which represent three dimensional plots of the benchmark function, the conversion curves of the RSA and FRSA, and the search histories, average fitness values, and trajectories. According to Figure 12, compared to the RSA, the FRSA proposed in this article had better exploration and development capabilities, and achieved higher exploration accuracy.

**Figure 13:**
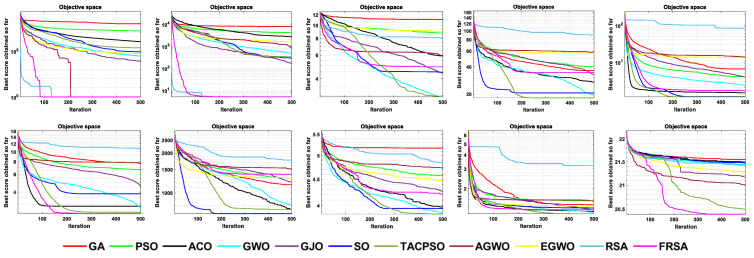
The convergence curves of the 11 algorithms on CEC 2019 functions.

**Figure 14:**
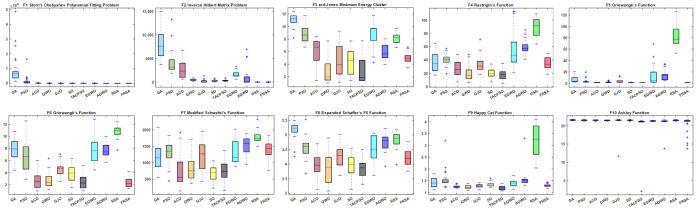
Boxplot analysis of CEC2019 benchmark functions.

All functions in the CEC 2019 are fixed dimensions. The design of this function set is more complex and can be used to demonstrate the robustness and universality of the proposed FRSA. Table 9 shows the results of solving the CEC 2019 using the FRSA and benchmark algorithms, including the Mean, Std, and Friedman test of 11 algorithms. Table 10 shows the FRSA’s Wilcoxon rank sum test results and those of the ten benchmark algorithms. According to Table 9, in the CEC 2019, the FRSA achieved optimal values for 4 functions, with the highest number among all 11 algorithms, in the Wilcoxon rank sum test and Friedman test. Wilcoxon’s rank sum test compared the FRSA with other algorithms, achieving a result of 58/18/24. The Friedman value showed the overall results of each algorithm in 10 functions. In the Friedman value, the FRSA achieved a result of 3.5500, ranking first in the Friedman rank. Both statistical methods proved that the FRSA achieved better results than the other algorithms in the CEC 2019 function. Figure 13 shows the iterative curves of the 11 algorithms in solving CEC 2019. Figure 14 presents a more comprehensive representation of the results of the 11 algorithms on the CEC 2019 function in the form of a boxplot.

This section compares the non-fixed dimensional and fixed dimensional functions from two different sets of functions with ten advanced algorithms to verify the performance of the FRSA. It is proved that the improvement strategies proposed in this article can effectively improve the performance of the original RSA and obtain better solutions. The proposed FRSA algorithm has a strong exploration ability and efficient space exploration ability and can effectively solve optimization problems in different dimensions.

## 5. Real-World Engineering Design Problems

In this section, the FRSA solves three engineering design problems: pressure vessel design [40,41], corrugated bulkhead design [42,43], and welded beam design [44]. Including multiple variables and multiple constraints, these problems are significant practical problems and are often used to verify the performance of heuristic algorithms. These engineering design problems have become a vital aspect of the practical application of meta-heuristic algorithms. To verify the performance of the FRSA more fairly, this section used ten advanced algorithms (GA, PSO, ACO, GWO, GJO, SO, TACPSO, AGWO, EGWO, and RSA) similar to the function testing section for testing.

### 5.1. Pressure Vessel Design

A pressure vessel is a closed container that can withstand pressure. The use of pressure vessels is pervasive, and they have an important position and role in many sectors, such as industry, civil service, military industry, and many fields of scientific research. In the design of a pressure vessel, under the constraints of four conditions, it is required to meet the production needs while maintaining the lowest total cost. The problem has four variables: the thickness of the shell $T_{s}\left(=x_{1}\right)$, the thickness of the head $T_{h}\left(=x_{2}\right)$, the inner radius $R\left(=x_{3}\right)$, and the length of the cylindrical section of the vessel, not including the head $L\left(=x_{4}\right)$. The mathematical model of the pressure vessel design is as follows:

$$\begin{array}{l} Min\;f(x)=0.6224x_{1}x_{3}x_{4}+1.7781x_{2}x_{3}^{2}+3.1661x_{1}^{2}x_{4}+19.84x_{1}^{2}x_{3}\\ Subject\;to\\ \;\;g_{1}(x)=-x_{1}+0.0193x_{3}\leq 0\\ \;\;g_{2}(x)=-x_{2}+0.00954x_{3}\leq 0\\ \;\;g_{3}(x)=-\pi x_{3}^{2}x_{4}-\frac{4}{3}\pi x_{3}^{2}+1296000\leq 0\\ \;\;g_{4}(x)=x_{4}-240\leq 0\\ where,\\ \;\;0\leq x_{1}\leq 99\\ \;\;0\leq x_{2}\leq 99\\ \;\;10\leq x_{3}\leq 200\\ \;\;10\leq x_{4}\leq 200 \end{array}$$

**Figure 15:**
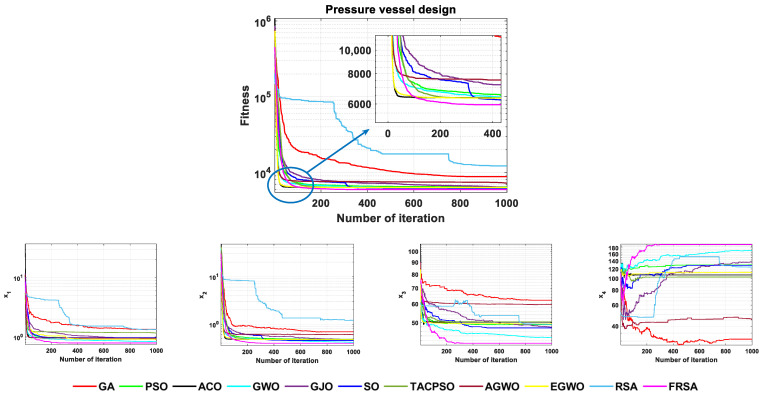
The convergence curves of 11 algorithms for the pressure vessel design problem.

The FRSA and ten other advanced algorithms proposed in this article were solved for the pressure vessel design problem. The minimum cost values required for pressure vessel production obtained by the 11 algorithms are shown in Table 11. According to the Table 11, the result obtained by the FRSA is $\vec{x}=\left\{0.77817,\;0.38465,\;40.32,\;200,\;5885.4\right\}$, which is the optimal result achieved among all 11 algorithms. To better demonstrate the optimization process of 11 algorithms in pressure vessel design problems, Figure 15 shows the convergence curves of the 11 algorithms, including the FRSA. It provides the corresponding change angles for each variable to reflect the trend of differences among the parameters during multi-parameter design. To verify the robustness of the algorithm on this issue, statistical analysis was also conducted, and the relevant statistical analysis data are shown in Table 12. Among them, the unit of time was seconds per experiment, that is, the average running time of each algorithm in a single experiment. The Wilcoxson rank sum test counted the results of the FRSA compared with other algorithms, and the FRSA achieved a result of 9/1/0. Through the corresponding convergence curve and statistical analysis, the FRSA converged faster and had higher accuracy and obvious advantages compared to the other algorithms.

### 5.2. Corrugated Bulkhead Design

A corrugated bulkhead is made of a pressed steel plate, and then it is bent to replace the function of the stiffener. In the corrugated bulkhead design problem, the minimum weight is required under the constraints of six conditions. The issue has four variables, which are the width ($x_{1}$), depth ($x_{2}$), length ($x_{3}$), and plate thickness ($x_{4}$). The mathematical model of the corrugated bulkhead design is as follows:

$$\begin{array}{l} Min\;f(x)=\frac{5.885x_{4}\left(x_{1}+x_{3}\right)}{x_{1}+\sqrt{\left|x_{3}^{2}-x_{2}^{2}\right|}}\\ Subject\;to\\ \;\;g_{1}(x)=-x_{4}x_{2}\left(0.4x_{1}+\frac{x_{3}}{6}\right)+8.94\left(x_{1}+\sqrt{\left|x_{3}^{2}-x_{2}^{2}\right|}\right)\leq 0\\ \;\;g_{2}(x)=-x_{4}x_{2}^{2}\left(0.3x_{1}+\frac{x_{3}}{12}\right)+2.2{\left(8.94\left(x_{1}+\sqrt{\left|x_{3}^{2}-x_{2}^{2}\right|}\right)\right)}^{\frac{4}{3}}\leq 0\\ \;\;g_{3}(x)=-x_{4}+0.0156x_{1}+0.15\leq 0\\ \;\;g_{4}(x)=-x_{4}+0.0156x_{3}+0.15\leq 0\\ \;\;g_{5}(x)=-x_{4}+1.05\leq 0\\ \;\;g_{6}(x)=-x_{3}+x_{2}\leq 0\\ where,\\ \;\;0\leq x_{1},x_{2},x_{3}\leq 100\;\;0\leq x_{4}\leq 5 \end{array}$$

**Figure 16:**
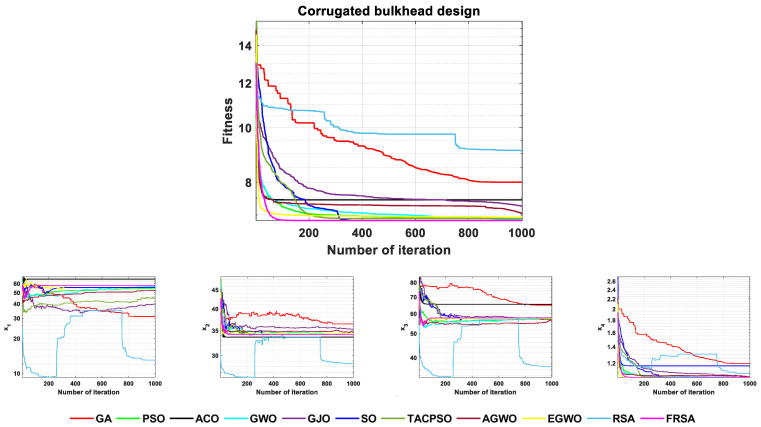
The convergence curves of 11 algorithms for the corrugated bulkhead design problem.

The FRSA and ten other advanced algorithms proposed in this article were solved for the corrugated bulkhead design problem. The corrugated bulkhead design values obtained by the 11 algorithms are shown in Table 13. According to the Table 13, the result obtained by the FRSA is $\vec{x}=\left\{57.692,\;34.148,\;57.692,\;1.05,\;6.8430\right\}$. Among all 11 algorithms, the FRSA achieved the best result. To better demonstrate the optimization process of the 11 algorithms in the corrugated bulkhead design problem, Figure 16 shows the convergence curves of the 11 algorithms, including the FRSA. It provides the corresponding change angles for each variable to reflect the trend of differences among the parameters during multi-parameter design. To verify the robustness of the algorithm on this issue, statistical analysis was also conducted, and the relevant statistical analysis results are shown in Table 14. The Wilcoxson rank sum test counted the results of the FRSA compared with the other algorithms, and the FRSA achieved a result of 9/0/1. Through the corresponding convergence curve and statistical analysis, the FRSA converged faster, had higher accuracy, and had obvious advantages compared to the other algorithms.

### 5.3. Welded Beam Design

A welded beam is a simplified model obtained for the convenience of calculation and analysis in material mechanics. One end of a cantilever beam is fixed support, and the other is free. This problem is a structural engineering design problem related to the weight optimization of square-section cantilever beams. The beams consist of five hollow blocks with constant thickness. The mathematical description of the welded beam design problem is as follows:

$$\begin{array}{l} Min\;f(x)=0.0624\left(x_{1}+x_{2}+x_{3}+x_{4}+x_{5}\right)\\ Subject\;to\\ \;\;g_{1}(x)=\frac{61}{x_{1}^{3}}+\frac{37}{x_{2}^{3}}+\frac{19}{x_{3}^{3}}+\frac{7}{x_{4}^{3}}+\frac{1}{x_{5}^{3}}\leq 0\\ where,\\ \;\;0.01\leq x_{1},x_{2},x_{3},x_{4},x_{5}\leq 100 \end{array}$$

**Figure 17:**
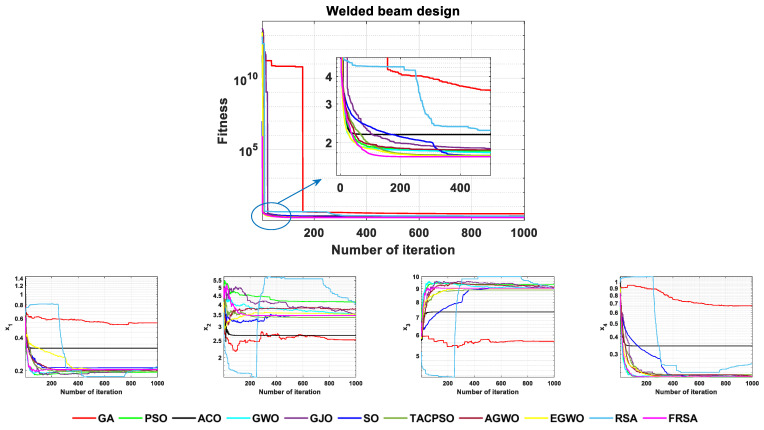
Convergence curves for the welded beam design problem.

The FRSA and ten other advanced algorithms proposed in this article were solved for the welded beam design problem. The values of the welded beam design obtained by the 11 algorithms are shown in Table 15. According to the Table 15, the result obtained by the FRSA is $\vec{x}=\left\{0.20573,\;3.4705,\;9.0366,\;0.20573,\;1.7249\right\}$. Among all 11 algorithms, the FRSA achieved the best result. To better demonstrate the optimization process of the 11 algorithms in the welded beam design problem, Figure 17 shows the convergence curves of the 11 algorithms, including the FRSA. It provides the corresponding change angles for each variable to reflect the trend of differences among the parameters during multi-parameter design. To verify the robustness of the algorithm on this issue, statistical analysis was also conducted, and the relevant statistical analysis results are shown in Table 16. The Wilcoxson rank sum test counted the results of the FRSA compared with the other algorithms, and FRSA achieved a result of 9/1/0. Through the corresponding convergence curve and statistical analysis, the FRSA converged faster, had higher accuracy, and had obvious advantages compared to the other algorithms.

## 6. Conclusions and Future Work

To improve the global optimization ability of the RSA, inspired by the different search horizons of different flying heights of natural creatures, this paper proposes a reptile algorithm considering different flying sizes based on the original RSA. In the exploration phase, introducing the different flight altitude abilities of two animals, the northern goshawk and the African vulture, enables reptiles to have better search horizons, improve their global search ability, and reduce the probability of falling into local optima during the exploration phase. In the exploration phase, a new *DF* is proposed to improve the algorithm’s convergence speed and optimization accuracy. To evaluate the effectiveness of the proposed FRSA, 33 benchmark functions were used for testing, including 13 non-fixed dimensional functions and 20 fixed dimensional functions. Among them, three different dimensions (30, 100, 500) were selected for the non-fixed dimensional functions for testing. The experimental and statistical results indicate that the FRSA has excellent performance and has certain advantages in accuracy, convergence speed, and stability compared to the ten most advanced algorithms. Furthermore, the FRSA was applied to solve three engineering optimization problems, and the results and comparison proved the algorithm’s effectiveness in solving practical problems.

In summary, the FRSA proposed in this article has good convergence accuracy, fast convergence speed, and good optimization performance. Through the testing of fixed and non-fixed dimensional functions and the validation of practical optimization problems, it has been proven that the proposed method can adapt to a wide range of optimization problems, and the algorithm’s robustness has been verified. In later research, the focus will be on evolving the proposed algorithm towards multi-objective optimization, such as path planning, workshop scheduling, and other fields, so that the proposed algorithm can generate more excellent value in practical life.

## Figures and Tables

**Table 1:** The classic function set.

Function	Dim	Range	$F_{min}$	Type
$f_{1}(x)=\sum_{i=1}^{n}x_{i}^{2}$	30,100,500	[−100, 100]	0	Unimodal
$f_{2}(x)=\sum_{i=1}^{n}\left|x_{i}\right|+\prod_{i=1}^{n}\left|x_{i}\right|$	30,100,500	[−1.28, 1.28]	0	Unimodal
$f_{3}(x)=\sum_{i=1}^{n}{\left(\sum_{j-1}^{i}x_{j}\right)}^{2}$	30,100,500	[−100, 100]	0	Unimodal
$f_{4}(x)=\max_{i}\left\{\left|x_{i}\right|,1⩽i⩽n\right\}$	30,100,500	[−100, 100]	0	Unimodal
$f_{5}(x)=\sum_{i=1}^{n-1}\left[100{\left(x_{i+1}-x_{i}^{2}\right)}^{2}+{\left(x_{i}-1\right)}^{2}\right]$	30,100,500	[−30, 30]	0	Unimodal
$f_{6}\left(x\right)=\overset{n}{\underset{i=1}{\sum }}{\left[x_{i}+0.5\right]}^{2}$	30,100,500	[−100, 100]	0	Unimodal
$f_{7}(x)=\sum_{i=1}^{n}ix_{i}^{4}+random[0,1]$	30,100,500	[−1.28, 1.28]	0	Unimodal
$f_{8}(x)=\sum_{i=1}^{n}-x_{i}\sin(\sqrt{\left|x_{i}\right|})$	30,100,500	[−500, 500]	−418.9829 × n	Multimodal
$f_{9}(x)=\sum_{i=1}^{n}\left[x_{i}^{2}-10\cos\left(2\pi x_{i}\right)+10\right]$	30,100,500	[−5.12, 5.12]	0	Multimodal
$\begin{array}{l} f_{10}(x)=-20\exp(-0.2\sqrt{\frac{1}{n}\sum_{i=1}^{n}x_{i}^{2}})-\\ \exp\left(\frac{1}{n}\sum_{i=1}^{n}\cos\left(2\pi x_{i}\right)\right)+20+e \end{array}$	30,100,500	[−32, 32]	0	Multimodal
$f_{11}(x)=\frac{1}{4000}\sum_{i=1}^{n}x_{i}^{2}-\prod_{i=1}^{n}\cos\left(\frac{x_{i}}{\sqrt{i}}\right)+1$	30,100,500	[−600, 600]	0	Multimodal
$\begin{array}{l} f_{12}\left(x\right)=\frac{\pi }{n}\left\{\begin{array}{l} 10\sin(\pi \mathrm{yi})+\sum_{i=1}^{n-1}{\left(y_{i}-1\right)}^{2}\left[1+10\sin^{2}(\pi y_{i+1})\right]\\ +{\left(y_{n}-1\right)}^{2} \end{array}\right\}+\\ \sum_{i=1}^{n}u(xi,10,100,4)\\ y_{i}=1+\frac{x_{i}+1}{4}\\ u(xi,a,k,m)=\left\{\begin{array}{l} k{\left(x_{i}-a\right)}^{m},x_{i}>a\\ 0,-a<x_{i}<a\\ k{\left(-x_{i}-a\right)}^{m},x_{i}<-a \end{array}\right\} \end{array}$	30,100,500	[−50, 50]	0	Multimodal
$\begin{array}{l} f_{13}(x)=0.1\left\{\begin{array}{l} \sin^{2}\left(3\pi x_{1}\right)+\sum_{i=1}^{n}{\left(x_{i}-1\right)}^{2}\left[1+\sin^{2}\left(3\pi x_{i}+1\right)\right]\\ +{\left(x_{n}-1\right)}^{2}\left[1+\sin^{2}\left(2\pi x_{n}\right)\right] \end{array}\right\}+\\ \sum_{i=1}^{n}u\left(x_{i},5,100,4\right) \end{array}$	30,100,500	[−50, 50]	0	Multimodal
$f_{14}(x)={\left(\frac{1}{500}+\sum_{j=1}^{25}\frac{1}{j+\sum_{i=1}^{2}{\left(x_{i}-a_{ij}\right)}^{6}}\right)}^{-1}$	2	[−65.536, 65.536]	1	Multimodal
$f_{15}(x)=\sum_{i=1}^{11}{\left[a_{i}-\frac{x_{1}\left(b_{i}^{2}+b_{i}x_{2}\right)}{b_{i}^{2}+b_{i}x_{3}+x_{4}}\right]}^{2}$	4	[−5, 5]	0.0003	Multimodal
$f_{16}(x)=4x_{1}^{2}-2.1x_{1}^{4}+\frac{1}{3}x_{1}^{6}+x_{1}x_{2}-4x_{2}^{2}+4x_{2}^{4}$	2	[−5, 5]	−1.0316	Multimodal
$f_{17}(x)={\left(x_{2}-\frac{5.1}{4\pi^{2}}x_{1}^{2}+\frac{5}{\pi }x_{1}-6\right)}^{2}+10\left(1-\frac{1}{8\pi }\right)\cos x_{1}+10$	2	[−5, 5]	0.398	Multimodal
$\begin{array}{l} f_{18}(x)=\left[1+{\left(x_{1}+x_{2}+1\right)}^{2}\left(19-14x_{1}+3x_{1}^{2}-14x_{2}+6x_{1}x_{2}+3x_{2}^{2}\right)\right]\\ \times \left[30+{\left(2x_{1}-3x_{2}\right)}^{2}\times \left(18-32x_{1}+12x_{1}^{2}+48x_{2}-36x_{1}x_{2}+27x_{2}^{2}\right)\right] \end{array}$	2	[−2, 2]	3	Multimodal
$f_{19}(x)=-\sum_{i=1}^{4}c_{i}\exp\left(-\sum_{j=1}^{3}a_{ij}{\left(x_{j}-p_{ij}\right)}^{2}\right)$	3	[0, 1]	−3.86	Multimodal
$f_{\begin{array}{l} 20 \end{array}}(x)=-\sum_{i=1}^{4}c_{i}\exp\left(-\sum_{j=1}^{6}a_{ij}{\left(x_{j}-p_{ij}\right)}^{2}\right)$	6	[0, 1]	−3.32	Multimodal
$f_{\begin{array}{l} 21 \end{array}}(x)=-\sum_{i=1}^{5}{\left[\left(X-a_{i}\right){\left(X-a_{i}\right)}^{T}+c_{i}\right]}^{-1}$	4	[0, 10]	−10.1532	Multimodal
$f_{22}(x)=-\sum_{i=1}^{7}{\left[\left(X-a_{i}\right){\left(X-a_{i}\right)}^{T}+c_{i}\right]}^{-1}$	4	[0, 10]	−10.4029	Multimodal
$f_{23}(x)=-\sum_{i=1}^{10}{\left[\left(X-a_{i}\right){\left(X-a_{i}\right)}^{T}+c_{i}\right]}^{-1}$	4	[0, 10]	−10.5364	Multimodal

**Table 2:** The CEC 2019 set.

No.	Functions	Dim	Range	$F_{i}^{*}=F_{i}\left(X^{*}\right)$
F1	Storn’s Chebyshev Polynomial Fitting Problem	9	[−8192, 8192]	1
F2	Inverse Hilbert Matrix Problem	16	[−16,384, 16,384]	1
F3	Lennard–Jones Minimum Energy Cluster	18	[−4, 4]	1
F4	Rastrigin’s Function	10	[−100, 100]	1
F5	Griewangk’s Function	10	[−100, 100]	1
F6	Weierstrass Function	10	[−100, 100]	1
F7	Modified Schwefel’s Function	10	[−100, 100]	1
F8	Expanded Schaffer’s F6 Function	10	[−100, 100]	1
F9	Happy Cat Function	10	[−100, 100]	1
F10	Ackley Function	10	[−100, 100]	1

**Table 3:** Parameter settings for algorithms.

Algorithms	Parameters and Assignments
GA	α∈[−0.5, 1.5]
PSO	$c_{1}=2$, $c_{2}=2$, $W_{\min}=0.2$, $W_{\max}=0.9$
ACO	α=1, β=2, ρ=0.05
GWO	a=2(linearly decreases over iterations), $r_{1}\in [0,1]$, $r_{2}\in [0,1]$
GJO	a=1.5(linearly decreases over iterations)
SO	a=2(linearly decreases over iterations)
TACPSO	$c_{1}=2$, $c_{2}=2$, $W_{\min}=0.2$, $W_{\max}=0.9$
AGWO	B=0.8, a=2(linearly decreases over iterations)
EGWO	a=2(linearly decreases over iterations), $r_{1}\in [0,1]$, $r_{2}\in [0,1]$
RSA	ε=0.1, ω=0.1
FRSA	$\varepsilon =0.1,\;\theta =2.5,\;L_{1}=0.8,\;L_{2}=0.2,\;$

**Table 4:** Results and comparison of 11 algorithms on 13 classic functions with Dim = 30.

F(x)		GA	PSO	ACO	GWO	GJO	SO	TACPSO	AGWO	EGWO	RSA	FRSA
F1	Mean	2.0706 × 10^4^	3.3853 × 10^2^	4.5737 × 10^−3^	1.0329 × 10^−27^	1.7311 × 10^−54^	3.9891 × 10^−94^	1.5111 × 10^−1^	3.2767 × 10^−317^	1.2009 × 10^−30^	**0.0000 × 10^0^**	**0.0000 × 10^0^**
	Std	7.1489 × 10^3^	1.6168 × 10^2^	6.7589 × 10^−3^	1.0808 × 10^−27^	4.1785 × 10^−54^	1.0339 × 10^−93^	2.3348 × 10^−1^	**0.0000 × 10^0^**	3.8756 × 10^−30^	**0.0000 × 10^0^**	**0.0000 × 10^0^**
F2	Mean	5.6471 × 10^1^	1.7592 × 10^1^	2.5207 × 10^−3^	1.0724 × 10^−16^	2.0077 × 10^−32^	1.8981 × 10^−42^	1.5195 × 10^0^	6.1333 × 10^−175^	8.6619 × 10^−20^	**0.0000 × 10^0^**	**0.0000 × 10^0^**
	Std	9.9694 × 10^0^	9.9392 × 10^0^	1.9247 × 10^−3^	8.1353 × 10^−17^	2.6567 × 10^−32^	7.8124 × 10^−42^	3.0452 × 10^0^	**0.0000 × 10^0^**	2.0027 × 10^−19^	**0.0000 × 10^0^**	**0.0000 × 10^0^**
F3	Mean	5.2325 × 10^4^	8.7587 × 10^3^	3.2509 × 10^4^	1.0617 × 10^−5^	8.0928 × 10^−18^	8.5384 × 10^−56^	1.1348 × 10^3^	5.2178 × 10^−264^	1.2199 × 10^−3^	**0.0000 × 10^0^**	**0.0000 × 10^0^**
	Std	1.5868 × 10^4^	5.3330 × 10^3^	7.1037 × 10^3^	2.7063 × 10^−5^	2.6301 × 10^−17^	3.6611 × 10^−55^	1.1917 × 10^3^	**0.0000 × 10^0^**	4.0506 × 10^−3^	**0.0000 × 10^0^**	**0.0000 × 10^0^**
F4	Mean	7.0290 × 10^1^	1.0057 × 10^1^	8.3925 × 10^1^	7.6327 × 10^−7^	5.5119 × 10^−16^	5.6706 × 10^−40^	9.7094 × 10^0^	8.5240 × 10^−155^	3.5666 × 10^−1^	**0.0000 × 10^0^**	**0.0000 × 10^0^**
	Std	7.2884 × 10^0^	2.6342 × 10^0^	1.1652 × 10^1^	8.4243 × 10^−7^	1.3025 × 10^−15^	1.9765 × 10^−39^	3.4154 × 10^0^	4.3706 × 10^−154^	1.3297 × 10^0^	**0.0000 × 10^0^**	**0.0000 × 10^0^**
F5	Mean	2.1143 × 10^7^	1.3458 × 10^4^	6.3852 × 10^2^	2.6950 × 10^1^	2.7744 × 10^1^	2.0242 × 10^1^	4.2784 × 10^2^	2.8334 × 10^1^	2.7928 × 10^1^	1.7547 × 10^1^	**9.0588 × 10^−29^**
	Std	1.5073 × 10^7^	9.7957 × 10^3^	9.3899 × 10^2^	7.1489 × 10^−1^	7.5092 × 10^−1^	1.1160 × 10^1^	9.0541 × 10^2^	3.9161 × 10^−1^	8.8237 × 10^−1^	1.4272 × 10^1^	**1.3586 × 10^−29^**
F6	Mean	2.2120 × 10^4^	3.3844 × 10^2^	**2.8991 × 10^−3^**	6.9336 × 10^−1^	2.5998 × 10^0^	7.4686 × 10^−1^	2.8608 × 10^−1^	5.1108 × 10^0^	3.1744 × 10^0^	6.9887 × 10^0^	9.3967 × 10^−3^
	Std	8.1756 × 10^3^	1.3189 × 10^2^	**4.3952 × 10^−3^**	3.2769 × 10^−1^	4.5246 × 10^−1^	7.2966 × 10^−1^	7.1367 × 10^−1^	3.2531 × 10^−1^	6.9967 × 10^−1^	4.0996 × 10^−1^	7.3219 × 10^−3^
F7	Mean	1.4246 × 10^1^	1.4084 × 10^0^	9.2893 × 10^−2^	2.1075 × 10^−3^	5.1434 × 10^−4^	2.9363 × 10^−4^	8.4275 × 10^−2^	**1.2253 × 10^−4^**	7.9773 × 10^−3^	1.2720 × 10^−4^	3.2019 × 10^−4^
	Std	6.4862 × 10^0^	5.8085 × 10^0^	3.6308 × 10^−2^	1.4913 × 10^−3^	3.3543 × 10^−4^	2.2856 × 10^−4^	3.3336 × 10^−2^	**9.7020 × 10^−5^**	4.0919 × 10^−3^	1.4087 × 10^−4^	3.1313 × 10^−4^
F8	Mean	−2.1820 × 10^3^	−8.0517 × 10^3^	−7.2210 × 10^3^	−5.8586 × 10^3^	−4.3233 × 10^3^	**−1.248 × 10^4^**	−8.6030 × 10^3^	−2.7317 × 10^3^	−6.5965 × 10^3^	−5.4035 × 10^3^	−1.1553 × 10^4^
	Std	4.0040 × 10^2^	9.6639 × 10^2^	1.0003 × 10^3^	7.5792 × 10^2^	1.2048 × 10^3^	**2.3899 × 10^2^**	4.6512 × 10^2^	4.6201 × 10^2^	7.6715 × 10^2^	3.1866 × 10^2^	1.6853 × 10^3^
F9	Mean	2.5863 × 10^2^	2.0098 × 10^2^	2.4292 × 10^2^	1.8876 × 10^0^	**0.0000 × 10^0^**	5.2470 × 10^0^	7.3533 × 10^1^	**0.0000 × 10^0^**	1.5967 × 10^2^	**0.0000 × 10^0^**	**0.0000 × 10^0^**
	Std	4.5208 × 10^1^	2.1856 × 10^1^	2.2226 × 10^1^	2.5924 × 10^0^	**0.0000 × 10^0^**	1.2881 × 10^1^	1.8901 × 10^1^	**0.0000 × 10^0^**	3.8336 × 10^1^	**0.0000 × 10^0^**	**0.0000 × 10^0^**
F10	Mean	1.9867 × 10^1^	5.3154 × 10^0^	1.2859 × 10^1^	1.0297 × 10^−13^	7.2831 × 10^−15^	2.8853 × 10^−1^	2.2423 × 10^0^	1.7171 × 10^−15^	1.9107 × 10^−1^	**8.8818 × 10^−16^**	**8.8818 × 10^−16^**
	Std	4.6960 × 10^−1^	1.0010 × 10^0^	9.8810 × 10^0^	1.8565 × 10^−14^	1.4454 × 10^−15^	7.5143 × 10^−1^	7.3942 × 10^−1^	1.5283 × 10^−15^	7.3243 × 10^−1^	**0.0000 × 10^0^**	**0.0000 × 10^0^**
F11	Mean	1.8735 × 10^2^	4.0848 × 10^0^	1.7211 × 10^−1^	4.9998 × 10^−3^	**0.0000 × 10^0^**	9.1944 × 10^−2^	1.3227 × 10^−1^	**0.0000 × 10^0^**	1.1550 × 10^−2^	**0.0000 × 10^0^**	**0.0000 × 10^0^**
	Std	6.6774 × 10^1^	1.8224 × 10^0^	2.7165 × 10^−1^	8.7540 × 10^−3^	**0.0000 × 10^0^**	1.7896 × 10^−1^	1.5411 × 10^−1^	**0.0000 × 10^0^**	2.1161 × 10^−2^	**0.0000 × 10^0^**	**0.0000 × 10^0^**
F12	Mean	1.7475 × 10^7^	5.9962 × 10^0^	3.2016 × 10^0^	4.7372 × 10^−2^	2.1168 × 10^−1^	1.2141 × 10^−1^	1.7178 × 10^0^	6.7014 × 10^−1^	3.1555 × 10^0^	1.2588 × 10^0^	**6.1299 × 10^−4^**
	Std	2.4583 × 10^7^	3.0819 × 10^0^	5.8093 × 10^0^	3.6729 × 10^−2^	6.8287 × 10^−2^	2.4035 × 10^−1^	1.6616 × 10^0^	1.4300 × 10^−1^	3.1014 × 10^0^	3.4982 × 10^−1^	**4.8674 × 10^−4^**
F13	Mean	5.7420 × 10^7^	2.8474 × 10^1^	2.2313 × 10^0^	6.8191 × 10^−1^	1.7212 × 10^0^	4.8266 × 10^−1^	4.1897 × 10^0^	2.5629 × 10^0^	2.6787 × 10^0^	4.1579 × 10^−1^	**3.8688 × 10^−31^**
	Std	4.5158 × 10^7^	2.9526 × 10^1^	5.0535 × 10^0^	2.5619 × 10^−1^	2.4044 × 10^−1^	6.9409 × 10^−1^	4.8206 × 10^0^	8.7892 × 10^−2^	5.8772 × 10^−1^	8.3308 × 10^−1^	**2.0585 × 10^−31^**
Friedman value		1.0423 × 10^1^	9.2692 × 10^0^	8.3846 × 10^0^	5.1538 × 10^0^	4.6923 × 10^0^	4.7308 × 10^0^	7.5385 × 10^0^	3.5385 × 10^0^	6.8077 × 10^0^	3.2500 × 10^0^	**2.2115 × 10^0^**
Friedman rank		11	10	9	6	4	5	8	3	7	2	**1**

**Table 5:** Results and comparison of 11 algorithms on 13 classic functions with Dim =100.

F(x)		GA	PSO	ACO	GWO	GJO	SO	TACPSO	AGWO	EGWO	RSA	FRSA
F1	Mean	2.2803 × 10^5^	4.8382 × 10^3^	1.1718 × 10^5^	1.2883 × 10^−12^	7.5690 × 10^−28^	7.3577 × 10^−82^	6.3258 × 10^3^	2.3337 × 10^−244^	2.9059 × 10^−16^	**0.0000 × 10^0^**	**0.0000 × 10^0^**
	Std	2.6407 × 10^4^	2.6614 × 10^3^	1.2634 × 10^4^	7.2714 × 10^−13^	2.0721 × 10^−27^	1.6214 × 10^−81^	1.9044 × 10^3^	**0.0000 × 10^0^**	4.3702 × 10^−16^	**0.0000 × 10^0^**	**0.0000 × 10^0^**
F2	Mean	1.3878 × 10^3^	7.9551 × 10^1^	1.0183 × 10^24^	4.0761 × 10^−8^	1.7716 × 10^−17^	1.1687 × 10^−35^	1.0765 × 10^2^	3.5171 × 10^−127^	2.1585 × 10^−10^	**0.0000 × 10^0^**	**0.0000 × 10^0^**
	Std	6.1674 × 10^3^	2.0688 × 10^1^	4.2616 × 10^24^	1.2357 × 10^−8^	1.9050 × 10^−17^	1.1341 × 10^−35^	2.4822 × 10^1^	1.9264 × 10^−126^	2.7251 × 10^−10^	**0.0000 × 10^0^**	**0.0000 × 10^0^**
F3	Mean	6.4151 × 10^5^	1.2058 × 10^5^	5.4194 × 10^5^	5.4654 × 10^2^	1.1960 × 10^0^	1.9856 × 10^−27^	7.9658 × 10^4^	9.4366 × 10^−220^	2.3095 × 10^4^	**0.0000 × 10^0^**	**0.0000 × 10^0^**
	Std	1.8635 × 10^5^	7.0068 × 10^4^	5.8785 × 10^4^	5.7433 × 10^2^	5.0520 × 10^0^	1.0876 × 10^−26^	1.7856 × 10^4^	**0.0000 × 10^0^**	1.5626 × 10^4^	**0.0000 × 10^0^**	**0.0000 × 10^0^**
F4	Mean	9.4654 × 10^1^	2.1438 × 10^1^	9.7253 × 10^1^	1.3792 × 10^0^	5.4031 × 10^0^	1.1115 × 10^−36^	4.4837 × 10^1^	1.4570 × 10^−130^	7.1629 × 10^1^	**0.0000 × 10^0^**	**0.0000 × 10^0^**
	Std	1.8813 × 10^0^	4.9340 × 10^0^	1.1638 × 10^0^	1.5201 × 10^0^	8.6012 × 10^0^	1.6885 × 10^−36^	3.1827 × 10^0^	5.4253 × 10^−130^	8.6435 × 10^0^	**0.0000 × 10^0^**	**0.0000 × 10^0^**
F5	Mean	8.5046 × 10^8^	5.2446 × 10^5^	1.0445 × 10^9^	9.7690 × 10^1^	9.8283 × 10^1^	6.4281 × 10^1^	3.2768 × 10^6^	9.8749 × 10^1^	9.8175 × 10^1^	9.8988 × 10^1^	**3.8975 × 10^−28^**
	Std	1.4410 × 10^8^	4.6652 × 10^5^	2.9092 × 10^8^	7.8639 × 10^−1^	4.8343 × 10^−1^	4.1497 × 10^1^	2.0490 × 10^6^	2.4079 × 10^−1^	6.4582 × 10^−1^	3.7169 × 10^−3^	**2.2620 × 10^−29^**
F6	Mean	2.1753 × 10^5^	3.9481 × 10^3^	1.1119 × 10^5^	1.0013 × 10^1^	1.6765 × 10^1^	1.4058 × 10^1^	6.3639 × 10^3^	2.2476 × 10^1^	1.4930 × 10^1^	2.4607 × 10^1^	**4.1033 × 10^−2^**
	Std	2.1653 × 10^4^	1.5048 × 10^3^	1.1425 × 10^4^	1.2537 × 10^0^	7.1104 × 10^−1^	1.0657 × 10^1^	2.8579 × 10^3^	3.1181 × 10^−1^	1.0606 × 10^0^	2.0760 × 10^−1^	**2.9663 × 10^−2^**
F7	Mean	1.2316 × 10^3^	5.3044 × 10^1^	8.4073 × 10^2^	7.4525 × 10^−3^	1.2061 × 10^−3^	2.2315 × 10^−4^	8.4554 × 10^0^	2.5098 × 10^−4^	2.9401 × 10^−2^	**1.1850 × 10^−4^**	2.4755 × 10^−4^
	Std	2.2446 × 10^2^	8.5484 × 10^1^	3.3353 × 10^2^	2.8388 × 10^−3^	5.1273 × 10^−4^	2.4369 × 10^−4^	4.9534 × 10^0^	2.3744 × 10^−4^	1.2773 × 10^−2^	**9.1632 × 10^−5^**	2.2133 × 10^−4^
F8	Mean	−4.1683 × 10^3^	−1.5010 × 10^4^	−1.5812 × 10^4^	−1.6026 × 10^4^	−9.1616 × 10^3^	**−4.1583 × 10^4^**	−2.2513 × 10^4^	−5.0509 × 10^3^	−1.7702 × 10^4^	−1.7056 × 10^4^	−3.6466 × 10^4^
	Std	9.7178 × 10^2^	2.6230 × 10^3^	2.7296 × 10^3^	2.4537 × 10^3^	4.2288 × 10^3^	**5.2761 × 10^2^**	1.9590 × 10^3^	9.0114 × 10^2^	1.4842 × 10^3^	7.6478 × 10^2^	7.1490 × 10^3^
F9	Mean	1.5280 × 10^3^	8.7473 × 10^2^	1.3949 × 10^3^	1.0982 × 10^1^	1.5158 × 10^−14^	1.4159 × 10^1^	4.6367 × 10^2^	**0.0000 × 10^0^**	8.3312 × 10^2^	**0.0000 × 10^0^**	**0.0000 × 10^0^**
	Std	6.4386 × 10^1^	8.6547 × 10^1^	4.5366 × 10^1^	8.3224 × 10^0^	5.7687 × 10^−14^	3.0090 × 10^1^	5.1780 × 10^1^	**0.0000 × 10^0^**	1.4958 × 10^2^	**0.0000 × 10^0^**	**0.0000 × 10^0^**
F10	Mean	2.0786 × 10^1^	9.0803 × 10^0^	2.0778 × 10^1^	1.1377 × 10^−7^	5.0271 × 10^−14^	4.4409 × 10^−15^	1.2679 × 10^1^	4.2040 × 10^−15^	8.4006 × 10^−2^	**8.8818 × 10^−16^**	**8.8818 × 10^−16^**
	Std	1.0176 × 10^−1^	2.4931 × 10^0^	4.0391 × 10^−2^	3.5782 × 10^−8^	9.8451 × 10^−15^	**0.0000 × 10^0^**	1.0867 × 10^0^	9.0135 × 10^−16^	4.6012 × 10^−1^	**0.0000 × 10^0^**	**0.0000 × 10^0^**
F11	Mean	1.9914 × 10^3^	3.5916 × 10^1^	1.0510 × 10^3^	5.6641 × 10^−3^	**0.0000 × 10^0^**	**0.0000 × 10^0^**	5.5387 × 10^1^	**0.0000 × 10^0^**	5.0051 × 10^−3^	**0.0000 × 10^0^**	**0.0000 × 10^0^**
	Std	2.1758 × 10^2^	1.3366 × 10^1^	1.1905 × 10^2^	1.2302 × 10^−2^	**0.0000 × 10^0^**	**0.0000 × 10^0^**	1.6313 × 10^1^	**0.0000 × 10^0^**	8.8528 × 10^−3^	**0.0000 × 10^0^**	**0.0000 × 10^0^**
F12	Mean	1.7624 × 10^9^	2.4562 × 10^3^	3.1606 × 10^9^	2.5960 × 10^−1^	6.0942 × 10^−1^	1.7964 × 10^−1^	1.4874 × 10^5^	1.0179 × 10^0^	1.0922 × 10^1^	1.2477 × 10^0^	**2.3383 × 10^−4^**
	Std	4.3233 × 10^8^	1.3099 × 10^4^	3.0994 × 10^8^	5.0711 × 10^−2^	7.7430 × 10^−2^	3.7770 × 10^−1^	4.4671 × 10^5^	6.5885 × 10^−2^	8.0541 × 10^0^	8.0783 × 10^−2^	**2.0208 × 10^−4^**
F13	Mean	3.4134 × 10^9^	4.4552 × 10^4^	5.6359 × 10^9^	6.8948 × 10^0^	8.3742 × 10^0^	2.1756 × 10^0^	2.4624 × 10^6^	9.6505 × 10^0^	2.6571 × 10^1^	9.6741 × 10^0^	**6.2822 × 10^−31^**
	Std	6.5988 × 10^8^	8.5336 × 10^4^	5.0013 × 10^8^	4.6552 × 10^−1^	2.3595 × 10^−1^	3.7113 × 10^0^	2.2076 × 10^6^	6.1528 × 10^−2^	3.9839 × 10^1^	5.8643 × 10^−1^	**1.8088 × 10^−31^**
Friedman value		1.0077 × 10^1^	8.4615 × 10^0^	9.6923 × 10^0^	5.4231 × 10^0^	5.0769 × 10^0^	3.8077 × 10^0^	8.3077 × 10^0^	3.5385 × 10^0^	6.6923 × 10^0^	2.9038 × 10^0^	**2.0192 × 10^0^**
Friedman rank		11	9	10	6	5	4	8	3	7	2	**1**

**Table 6:** Results and comparison of 11 algorithms on 13 classic functions with Dim = 500.

F(x)		GA	PSO	ACO	GWO	GJO	SO	TACPSO	AGWO	EGWO	RSA	FRSA
F1	Mean	1.5128 × 10^6^	3.9219 × 10^4^	1.5590 × 10^6^	1.8644 × 10^−3^	9.6545 × 10^−13^	7.1375 × 10^−71^	2.9775 × 10^5^	1.9542 × 10^−16^	6.1307 × 10^−6^	**0.0000 × 10^0^**	**0.0000 × 10^0^**
	Std	3.6434 × 10^4^	1.3201 × 10^4^	3.6597 × 10^4^	7.6449 × 10^−4^	9.7800 × 10^−13^	2.4182 × 10^−70^	1.9178 × 10^4^	1.0703 × 10^−15^	6.2289 × 10^−6^	**0.0000 × 10^0^**	**0.0000 × 10^0^**
F2	Mean	6.0554 × 10^226^	4.5845 × 10^2^	4.1585 × 10^268^	1.0881 × 10^−2^	6.4312 × 10^−9^	1.2654 × 10^−31^	6.3084 × 10^17^	9.6613 × 10^−12^	1.8407 × 10^−4^	**0.0000 × 10^0^**	**0.0000 × 10^0^**
	Std	Inf	1.2379 × 10^2^	Inf	1.7840 × 10^−3^	4.2103 × 10^−9^	1.7875 × 10^−31^	3.4548 × 10^18^	5.2623 × 10^−11^	1.4881 × 10^−4^	**0.0000 × 10^0^**	**0.0000 × 10^0^**
F3	Mean	2.1316 × 10^7^	2.8293 × 10^6^	1.3418 × 10^7^	3.1425 × 10^5^	5.1301 × 10^4^	8.2145 × 10^2^	2.0650 × 10^6^	1.2415 × 10^−5^	1.5987 × 10^6^	**0.0000 × 10^0^**	**0.0000 × 10^0^**
	Std	6.9901 × 10^6^	1.4370 × 10^6^	1.3908 × 10^6^	7.7943 × 10^4^	5.3267 × 10^4^	4.4993 × 10^3^	4.4012 × 10^5^	4.9557 × 10^−5^	2.9634 × 10^5^	**0.0000 × 10^0^**	**0.0000 × 10^0^**
F4	Mean	9.9161 × 10^1^	3.5159 × 10^1^	9.9451 × 10^1^	6.5006 × 10^1^	8.2792 × 10^1^	1.4350 × 10^−33^	7.0229 × 10^1^	9.2608 × 10^1^	9.6997 × 10^1^	**0.0000 × 10^0^**	**0.0000 × 10^0^**
	Std	2.3489 × 10^−1^	5.2254 × 10^0^	1.9881 × 10^−1^	4.1463 × 10^0^	4.3209 × 10^0^	1.9441 × 10^−33^	2.9160 × 10^0^	2.5175 × 10^1^	9.6498 × 10^−1^	**0.0000 × 10^0^**	**0.0000 × 10^0^**
F5	Mean	6.7974 × 10^9^	8.5251 × 10^6^	7.3446 × 10^9^	4.9803 × 10^2^	4.9826 × 10^2^	3.3551 × 10^2^	4.1692 × 10^8^	4.9892 × 10^2^	2.0683 × 10^5^	4.9899 × 10^2^	**2.3167 × 10^−27^**
	Std	2.6217 × 10^8^	5.6668 × 10^6^	3.3600 × 10^8^	3.5404 × 10^−1^	1.4870 × 10^−1^	2.1069 × 10^2^	3.9772 × 10^7^	6.8551 × 10^−2^	3.5928 × 10^5^	6.2629 × 10^−3^	**6.3915 × 10^−29^**
F6	Mean	1.5114 × 10^6^	3.5914 × 10^4^	1.5648 × 10^6^	9.1100 × 10^1^	1.1002 × 10^2^	6.6077 × 10^1^	2.9553 × 10^5^	1.2326 × 10^2^	1.0553 × 10^2^	1.2463 × 10^2^	**2.6280 × 10^−1^**
	Std	3.8311 × 10^4^	1.7013 × 10^4^	3.9734 × 10^4^	1.8331 × 10^0^	1.2181 × 10^0^	5.6041 × 10^1^	1.6752 × 10^4^	4.2145 × 10^−1^	1.6851 × 10^0^	2.1010 × 10^−1^	**2.2051 × 10^−1^**
F7	Mean	5.6877 × 10^4^	2.2092 × 10^3^	5.9688 × 10^4^	5.1280 × 10^−2^	6.5673 × 10^−3^	2.4772 × 10^−4^	5.5137 × 10^3^	4.1447 × 10^−3^	2.2992 × 10^0^	**1.6209 × 10^−4^**	2.8637 × 10^−4^
	Std	1.7801 × 10^3^	1.1383 × 10^3^	2.5418 × 10^3^	1.2074 × 10^−2^	3.9213 × 10^−3^	1.6724 × 10^−4^	1.0697 × 10^3^	2.4388 × 10^−3^	2.0536 × 10^0^	**1.9236 × 10^−4^**	2.5568 × 10^−4^
F8	Mean	−8.6802 × 10^3^	−3.6383 × 10^4^	−3.1971 × 10^4^	−5.3591 × 10^4^	−2.6579 × 10^4^	**−2.0786 × 10^5^**	−6.3227 × 10^4^	−1.0502 × 10^4^	−4.6206 × 10^4^	−6.1323 × 10^4^	−1.8676 × 10^5^
	Std	1.6314 × 10^3^	5.2307 × 10^3^	6.0899 × 10^3^	1.3793 × 10^4^	1.4002 × 10^4^	3.1591 × 10^3^	2.8055 × 10^3^	**1.2858 × 10^3^**	2.9204 × 10^3^	5.3327 × 10^3^	3.2489 × 10^4^
F9	Mean	8.6929 × 10^3^	4.4339 × 10^3^	8.8775 × 10^3^	7.5607 × 10^1^	7.3063 × 10^−12^	7.3183 × 10^0^	4.4337 × 10^3^	3.0028 × 10^−10^	5.0232 × 10^3^	**0.0000 × 10^0^**	**0.0000 × 10^0^**
	Std	1.2985 × 10^2^	5.4177 × 10^2^	1.0714 × 10^2^	2.1468 × 10^1^	2.8809 × 10^−12^	3.9910 × 10^1^	1.3537 × 10^2^	1.6447 × 10^−9^	1.1423 × 10^3^	**0.0000 × 10^0^**	**0.0000 × 10^0^**
F10	Mean	2.1105 × 10^1^	1.1398 × 10^1^	2.1018 × 10^1^	1.8561 × 10^−3^	3.1785 × 10^−8^	4.9146 × 10^−15^	1.8242 × 10^1^	2.6645 × 10^−15^	1.5959 × 10^−4^	**8.8818 × 10^−16^**	**8.8818 × 10^−16^**
	Std	2.8935 × 10^−2^	3.5690 × 10^0^	1.0087 × 10^−2^	3.7330 × 10^−4^	1.5956 × 10^−8^	1.2283 × 10^−15^	1.9259 × 10^−1^	1.8067 × 10^−15^	9.5884 × 10^−5^	**0.0000 × 10^0^**	**0.0000 × 10^0^**
F11	Mean	1.3426 × 10^4^	3.0914 × 10^2^	1.4057 × 10^4^	2.0278 × 10^−2^	1.0707 × 10^−13^	**0.0000 × 10^0^**	2.6951 × 10^3^	**0.0000 × 10^0^**	1.6181 × 10^−2^	**0.0000 × 10^0^**	**0.0000 × 10^0^**
	Std	3.5235 × 10^2^	9.0526 × 10^1^	3.4112 × 10^2^	4.1331 × 10^−2^	9.3215 × 10^−14^	**0.0000 × 10^0^**	1.5936 × 10^2^	**0.0000 × 10^0^**	3.4848 × 10^−2^	**0.0000 × 10^0^**	**0.0000 × 10^0^**
F12	Mean	1.7068 × 10^10^	2.0914 × 10^5^	1.8490 × 10^10^	7.6115 × 10^−1^	9.3858 × 10^−1^	5.1918 × 10^−2^	3.4826 × 10^8^	1.1681 × 10^0^	5.9375 × 10^7^	1.2016 × 10^0^	**2.1631 × 10^−4^**
	Std	7.0596 × 10^8^	3.1495 × 10^5^	6.2478 × 10^8^	7.5436 × 10^−2^	2.6207 × 10^−2^	2.0980 × 10^−1^	7.1676 × 10^7^	1.0173 × 10^−2^	6.2911 × 10^7^	2.9273 × 10^−3^	**2.1681 × 10^−4^**
F13	Mean	3.1399 × 10^10^	6.4059 × 10^6^	3.3112 × 10^10^	5.0441 × 10^1^	4.7911 × 10^1^	7.2088 × 10^0^	1.1450 × 10^9^	4.9797 × 10^1^	1.1536 × 10^7^	4.9921 × 10^1^	**2.0996 × 10^−30^**
	Std	1.3772 × 10^9^	8.1295 × 10^6^	1.3777 × 10^9^	1.4970 × 10^0^	3.2400 × 10^−1^	1.4763 × 10^1^	1.5104 × 10^8^	4.1931 × 10^−2^	1.7631 × 10^7^	3.9674 × 10^−2^	**9.4926 × 10^−32^**
Friedman value		9.6346 × 10^0^	8.0385 × 10^0^	9.9808 × 10^0^	5.9231 × 10^0^	5.1154 × 10^0^	3.5000 × 10^0^	8.1538 × 10^0^	4.3077 × 10^0^	6.7692 × 10^0^	2.6154 × 10^0^	**1.9615 × 10^0^**
Friedman rank		10	8	11	6	5	3	9	4	7	2	**1**

**Table 7:** Results and comparison of 11 algorithms on 10 classic functions with fixed dimensions.

F(x)		GA	PSO	ACO	GWO	GJO	SO	TACPSO	AGWO	EGWO	RSA	FRSA
F14	Mean	1.1036 × 10^0^	**9.9800 × 10^−1^**	2.8537 × 10^0^	5.0796 × 10^0^	5.3036 × 10^0^	1.0022 × 10^0^	1.0311 × 10^0^	6.4801 × 10^0^	7.7381 × 10^0^	4.1376 × 10^0^	9.9823 × 10^−1^
	Std	3.3201 × 10^−1^	**2.1481 × 10^−10^**	3.8575 × 10^0^	4.1695 × 10^0^	4.4384 × 10^0^	2.0981 × 10^−2^	1.8148 × 10^−1^	4.3221 × 10^0^	4.4611 × 10^0^	3.1646 × 10^0^	1.2224 × 10^−3^
F15	Mean	1.3902 × 10^−2^	1.0272 × 10^−2^	5.3931 × 10^−3^	3.0739 × 10^−3^	8.5798 × 10^−4^	6.0445 × 10^−4^	5.2544 × 10^−4^	1.4132 × 10^−3^	1.0979 × 10^−2^	1.7245 × 10^−3^	**4.1525 × 10^−4^**
	Std	1.0043 × 10^−2^	1.0209 × 10^−2^	8.4021 × 10^−3^	6.8994 × 10^−3^	2.0507 × 10^−3^	3.3346 × 10^−4^	4.1271 × 10^−4^	2.7639 × 10^−3^	2.3937 × 10^−2^	1.4282 × 10^−3^	**8.1372 × 10^−5^**
F16	Mean	−9.4538 × 10^−1^	**−1.0316 × 10^0^**	**−1.0316 × 10^0^**	**−1.0316 × 10^0^**	**−1.0316 × 10^0^**	**−1.0316 × 10^0^**	−1.0316 × 10^0^	−1.0306 × 10^0^	−1.0316 × 10^0^	−1.0305 × 10^0^	**−1.0316 × 10^0^**
	Std	1.1796 × 10^−1^	1.5212 × 10^−5^	6.7752 × 10^−16^	1.8976 × 10^−8^	2.5177 × 10^−7^	**5.2964 × 10^−16^**	5.9036 × 10^−16^	5.7742 × 10^−3^	5.6187 × 10^−9^	1.4232 × 10^−3^	1.8373 × 10^−13^
F17	Mean	4.0005 × 10^−1^	**3.9789 × 10^−1^**	**3.9789 × 10^−1^**	**3.9789 × 10^−1^**	**3.9789 × 10^−1^**	**3.9789 × 10^−1^**	**3.9789 × 10^−1^**	3.9794 × 10^−1^	3.9789 × 10^−1^	4.1970 × 10^−1^	**3.9789 × 10^−1^**
	Std	4.0846 × 10^−3^	1.4541 × 10^−5^	**0.0000 × 10^0^**	7.2876 × 10^−7^	9.0667 × 10^−6^	**0.0000 × 10^0^**	**0.0000 × 10^0^**	5.3183 × 10^−5^	5.9598 × 10^−7^	2.4368 × 10^−2^	**0.0000 × 10^0^**
F18	Mean	1.0596 × 10^1^	3.0002 × 10^0^	**3.0000 × 10^0^**	**3.0000 × 10^0^**	**3.0000 × 10^0^**	**3.0000 × 10^0^**	**3.0000 × 10^0^**	**3.0000 × 10^0^**	3.9001 × 10^0^	4.0014 × 10^0^	**3.0000 × 10^0^**
	Std	1.1443 × 10^1^	2.8621 × 10^−4^	**6.6995 × 10^−16^**	4.8544 × 10^−5^	8.5395 × 10^−6^	2.7088 × 10^−15^	2.1599 × 10^−15^	1.8450 × 10^−6^	4.9295 × 10^0^	5.4822 × 10^0^	3.7510 × 10^−15^
F19	Mean	−3.2754 × 10^0^	−3.8614 × 10^0^	**−3.8628 × 10^0^**	−3.8612 × 10^0^	−3.8581 × 10^0^	−3.8370 × 10^0^	**−3.8628 × 10^0^**	−3.8569 × 10^0^	−3.8618 × 10^0^	−3.7992 × 10^0^	**−3.8628 × 10^0^**
	Std	3.2324 × 10^−1^	2.9771 × 10^−3^	2.7101 × 10^−15^	2.6343 × 10^−3^	3.7740 × 10^−3^	1.4113 × 10^−1^	2.6117 × 10^−15^	2.6408 × 10^−3^	2.6029 × 10^−3^	6.3061 × 10^−2^	**2.0748 × 10^−15^**
F20	Mean	−1.4764 × 10^0^	−3.0759 × 10^0^	−3.2467 × 10^0^	−3.2796 × 10^0^	−3.0914 × 10^0^	−3.2982 × 10^0^	−3.2665 × 10^0^	−3.1263 × 10^0^	−3.2177 × 10^0^	−2.7566 × 10^0^	**−3.3213 × 10^0^**
	Std	4.8085 × 10^−1^	1.9536 × 10^−1^	5.8273 × 10^−2^	6.9288 × 10^−2^	1.3582 × 10^−1^	4.8370 × 10^−2^	6.0328 × 10^−2^	1.0519 × 10^−1^	9.9155 × 10^−2^	3.4506 × 10^−1^	**2.7018 × 10^−3^**
F21	Mean	−8.5022 × 10^−1^	−9.0585 × 10^0^	−5.9936 × 10^0^	−9.0574 × 10^0^	−7.7219 × 10^0^	**−1.0138 × 10^1^**	−6.8143 × 10^0^	−7.3462 × 10^0^	−6.2985 × 10^0^	−5.0552 × 10^0^	−1.0105 × 10^1^
	Std	5.1246 × 10^−1^	2.0337 × 10^0^	3.7255 × 10^0^	2.2621 × 10^0^	2.9320 × 10^0^	3.4059 × 10^−2^	3.4941 × 10^0^	2.9488 × 10^0^	3.1346 × 10^0^	**3.1204 × 10^−7^**	7.9343 × 10^−2^
F22	Mean	−1.0336 × 10^0^	−9.0891 × 10^0^	−7.4926 × 10^0^	−1.0401 × 10^1^	−9.8499 × 10^0^	−1.0290 × 10^1^	−7.1316 × 10^0^	−8.5041 × 10^0^	−7.1293 × 10^0^	−5.0877 × 10^0^	**−1.0402 × 10^1^**
	Std	4.4156 × 10^−1^	2.6893 × 10^0^	3.6556 × 10^0^	1.2043 × 10^−3^	1.6359 × 10^0^	2.5749 × 10^−1^	3.4330 × 10^0^	2.5694 × 10^0^	3.6624 × 10^0^	**8.0616 × 10^−7^**	4.1384 × 10^−3^
F23	Mean	−1.2002 × 10^0^	−9.0372 × 10^0^	−7.2815 × 10^0^	−9.9938 × 10^0^	−9.6040 × 10^0^	−1.0469 × 10^1^	−9.4877 × 10^0^	−8.6658 × 10^0^	−6.4546 × 10^0^	−5.1314 × 10^0^	**−1.0525 × 10^1^**
	Std	3.9772 × 10^−1^	2.6192 × 10^0^	3.8049 × 10^0^	2.0583 × 10^0^	2.4090 × 10^0^	1.4991 × 10^−1^	2.4300 × 10^0^	2.5916 × 10^0^	3.8996 × 10^0^	1.6091 × 10^−2^	**3.3992 × 10^−2^**
Friedman value		9.2000 × 10^0^	6.4000 × 10^0^	5.8250 × 10^0^	5.1500 × 10^0^	6.2000 × 10^0^	3.4250 × 10^0^	4.5250 × 10^0^	7.3000 × 10^0^	7.8500 × 10^0^	7.8000 × 10^0^	**2.3250 × 10^0^**
Friedman rank		11	7	5	4	6	2	3	8	10	9	**1**

**Table 8:** Statistical analysis results of Wilcoxon rank sum test of classic functions.

F(x)	Dim	GA	PSO	ACO	GWO	GJO	SO	TACPSO	AGWO	EGWO	RSA	Total
F1	30	1.2118 × 10^−12^	1.2118 × 10^−12^	1.2118 × 10^−12^	1.2118 × 10^−12^	1.2118 × 10^−12^	1.2118 × 10^−12^	1.2118 × 10^−12^	1.2118 × 10^−12^	1.2118 × 10^−12^	NaN	9/1/0
	100	1.2118 × 10^−12^	1.2118 × 10^−12^	1.2118 × 10^−12^	1.2118 × 10^−12^	1.2118 × 10^−12^	1.2118 × 10^−12^	1.2118 × 10^−12^	1.2118 × 10^−12^	1.9346 × 10^−10^	NaN	9/1/0
	500	1.2118 × 10^−12^	1.2118 × 10^−12^	1.2118 × 10^−12^	1.2118 × 10^−12^	1.2118 × 10^−12^	1.2118 × 10^−12^	1.2118 × 10^−12^	1.2118 × 10^−12^	1.2118 × 10^−12^	NaN	9/1/0
F2	30	1.2118 × 10^−12^	1.2118 × 10^−12^	1.2118 × 10^−12^	1.2118 × 10^−12^	1.2118 × 10^−12^	1.2118 × 10^−12^	1.2118 × 10^−12^	1.2118 × 10^−12^	1.2118 × 10^−12^	NaN	9/1/0
	100	1.2118 × 10^−12^	1.2118 × 10^−12^	1.2118 × 10^−12^	1.2118 × 10^−12^	1.2118 × 10^−12^	1.2118 × 10^−12^	1.2118 × 10^−12^	1.2118 × 10^−12^	1.2118 × 10^−12^	NaN	9/1/0
	500	1.2118 × 10^−12^	1.2118 × 10^−12^	1.2118 × 10^−12^	1.2118 × 10^−12^	1.2118 × 10^−12^	1.2118 × 10^−12^	1.2118 × 10^−12^	1.2118 × 10^−12^	1.2118 × 10^−12^	NaN	9/1/0
F3	30	1.2118 × 10^−12^	1.2118 × 10^−12^	1.2118 × 10^−12^	1.2118 × 10^−12^	1.2118 × 10^−12^	1.2118 × 10^−12^	1.2118 × 10^−12^	1.2118 × 10^−12^	1.2118 × 10^−12^	NaN	9/1/0
	100	1.2118 × 10^−12^	1.2118 × 10^−12^	1.2118 × 10^−12^	1.2118 × 10^−12^	1.2118 × 10^−12^	1.2118 × 10^−12^	1.2118 × 10^−12^	1.2118 × 10^−12^	4.5736 × 10^−12^	NaN	9/1/0
	500	1.2118 × 10^−12^	1.2118 × 10^−12^	1.2118 × 10^−12^	1.2118 × 10^−12^	1.2118 × 10^−12^	1.2118 × 10^−12^	1.2118 × 10^−12^	1.2118 × 10^−12^	1.2118 × 10^−12^	NaN	9/1/0
F4	30	1.2118 × 10^−12^	1.2118 × 10^−12^	1.2118 × 10^−12^	1.2118 × 10^−12^	1.2118 × 10^−12^	1.2118 × 10^−12^	1.2118 × 10^−12^	1.2118 × 10^−12^	1.2118 × 10^−12^	NaN	9/1/0
	100	1.2118 × 10^−12^	1.2118 × 10^−12^	1.2118 × 10^−12^	1.2118 × 10^−12^	1.2118 × 10^−12^	1.2118 × 10^−12^	1.2118 × 10^−12^	1.2118 × 10^−12^	1.2118 × 10^−12^	NaN	9/1/0
	500	1.2118 × 10^−12^	1.2118 × 10^−12^	1.2118 × 10^−12^	1.2118 × 10^−12^	1.2118 × 10^−12^	1.2118 × 10^−12^	1.2118 × 10^−12^	1.2118 × 10^−12^	1.2118 × 10^−12^	NaN	9/1/0
F5	30	3.0161 × 10^−11^	3.0161 × 10^−11^	3.0161 × 10^−11^	3.0161 × 10^−11^	3.0161 × 10^−11^	3.0161 × 10^−11^	3.0161 × 10^−11^	3.0161 × 10^−11^	3.0161 × 10^−11^	3.0161 × 10^−11^	10/0/0
	100	3.0161 × 10^−11^	3.0161 × 10^−11^	3.0161 × 10^−11^	3.0161 × 10^−11^	3.0161 × 10^−11^	3.0161 × 10^−11^	3.0161 × 10^−11^	3.0161 × 10^−11^	3.0161 × 10^−11^	3.0161 × 10^−11^	10/0/0
	500	3.0199 × 10^−11^	3.0199 × 10^−11^	3.0199 × 10^−11^	3.0199 × 10^−11^	3.0199 × 10^−11^	3.0199 × 10^−11^	3.0199 × 10^−11^	3.0199 × 10^−11^	3.0199 × 10^−11^	3.0199 × 10^−11^	10/0/0
F6	30	3.0199 × 10^−11^	3.0199 × 10^−11^	2.3168 × 10^−6^	3.0199 × 10^−11^	3.0199 × 10^−11^	1.0937 × 10^−10^	3.1573 × 10^−5^	3.0199 × 10^−11^	3.0199 × 10^−11^	3.0199 × 10^−11^	9/0/1
	100	3.0161 × 10^−11^	3.0161 × 10^−11^	3.0161 × 10^−11^	3.0161 × 10^−11^	3.0161 × 10^−11^	8.9934 × 10^−11^	3.0161 × 10^−11^	3.0161 × 10^−11^	3.0161 × 10^−11^	3.0161 × 10^−11^	10/0/0
	500	3.0199 × 10^−11^	3.0199 × 10^−11^	3.0199 × 10^−11^	3.0199 × 10^−11^	3.0199 × 10^−11^	4.9752 × 10^−11^	3.0199 × 10^−11^	3.0199 × 10^−11^	3.0199 × 10^−11^	3.0199 × 10^−11^	10/0/0
F7	30	3.0199 × 10^−11^	3.0199 × 10^−11^	3.0199 × 10^−11^	1.2057 × 10^−10^	1.0315 × 10^−2^	9.8231 × 10^−1^	3.0199 × 10^−11^	3.3384 × 10^−11^	3.5010 × 10^−3^	1.7666 × 10^−3^	7/1/2
	100	3.0161 × 10^−11^	3.0161 × 10^−11^	3.0161 × 10^−11^	3.0161 × 10^−11^	7.3803 × 10^−10^	6.7350 × 10^−1^	3.0199 × 10^−11^	3.0199 × 10^−11^	7.0617 × 10^−1^	2.4157 × 10^−2^	7/2/1
	500	3.0199 × 10^−11^	3.0199 × 10^−11^	3.0199 × 10^−11^	3.0199 × 10^−11^	3.0199 × 10^−11^	9.8231 × 10^−1^	3.0199 × 10^−11^	3.0199 × 10^−11^	3.4742 × 10^−10^	4.3584 × 10^−2^	8/1/1
F8	30	3.0199 × 10^−11^	1.2541 × 10^−7^	2.1947 × 10^−8^	4.1997 × 10^−10^	7.3891 × 10^−11^	1.3017 × 10^−3^	3.3681 × 10^−5^	2.2273 × 10^−9^	3.0199 × 10^−11^	2.6099 × 10^−10^	9/0/1
	100	3.0161 × 10^−11^	3.0161 × 10^−11^	3.0161 × 10^−11^	3.0161 × 10^−11^	3.0161 × 10^−11^	4.5146 × 10^−2^	1.4110 × 10^−9^	3.0199 × 10^−11^	3.0199 × 10^−11^	2.9878 × 10^−11^	9/0/1
	500	3.0199 × 10^−11^	3.0199 × 10^−11^	3.0199 × 10^−11^	3.0199 × 10^−11^	3.0199 × 10^−11^	4.0595 × 10^−2^	3.0199 × 10^−11^	3.0199 × 10^−11^	3.0199 × 10^−11^	3.0199 × 10^−11^	9/0/1
F9	30	1.2118 × 10^−12^	1.2118 × 10^−12^	1.2118 × 10^−12^	1.1378 × 10^−12^	NaN	1.9457 × 10^−9^	1.2118 × 10^−12^	1.2118 × 10^−12^	NaN	NaN	7/3/0
	100	1.2118 × 10^−12^	1.2118 × 10^−12^	1.2118 × 10^−12^	1.2118 × 10^−12^	1.6074 × 10^−1^	5.3750 × 10^−6^	1.2118 × 10^−12^	1.2118 × 10^−12^	NaN	NaN	7/3/0
	500	1.2118 × 10^−12^	1.2118 × 10^−12^	1.2118 × 10^−12^	1.2118 × 10^−12^	1.0956 × 10^−12^	4.1926 × 10^−2^	1.2118 × 10^−12^	1.2118 × 10^−12^	3.3371 × 10^−1^	NaN	8/2/0
F10	30	1.2118 × 10^−12^	1.2118 × 10^−12^	1.2118 × 10^−12^	1.1001 × 10^−12^	1.5479 × 10^−13^	1.2003 × 10^−13^	1.2118 × 10^−12^	5.3025 × 10^−13^	5.4660 × 10^−3^	NaN	9/1/0
	100	1.2118 × 10^−12^	1.2118 × 10^−12^	1.2118 × 10^−12^	1.2118 × 10^−12^	1.0171 × 10^−12^	1.6853 × 10^−14^	1.2118 × 10^−12^	1.2118 × 10^−12^	7.1518 × 10^−13^	NaN	9/1/0
	500	1.2118 × 10^−12^	1.2118 × 10^−12^	1.2118 × 10^−12^	1.2118 × 10^−12^	1.2118 × 10^−12^	8.6442 × 10^−14^	1.2118 × 10^−12^	1.2118 × 10^−12^	9.6506 × 10^−6^	NaN	9/1/0
F11	30	1.2118 × 10^−12^	1.2118 × 10^−12^	1.2118 × 10^−12^	2.7880 × 10^−3^	NaN	1.3702 × 10^−3^	1.2118 × 10^−12^	2.9343 × 10^−5^	NaN	NaN	8/2/0
	100	1.2118 × 10^−12^	1.2118 × 10^−12^	1.2118 × 10^−12^	1.2118 × 10^−12^	NaN	NaN	1.2118 × 10^−12^	5.8153 × 10^−9^	NaN	NaN	6/4/0
	500	1.2118 × 10^−12^	1.2118 × 10^−12^	1.2118 × 10^−12^	1.2118 × 10^−12^	1.2118 × 10^−12^	NaN	1.2118 × 10^−12^	1.2118 × 10^−12^	NaN	NaN	7/3/0
F12	30	1.5099 × 10^−11^	3.0199 × 10^−11^	3.0199 × 10^−11^	3.0199 × 10^−11^	3.0199 × 10^−11^	2.3897 × 10^−8^	3.0199 × 10^−11^	3.0199 × 10^−11^	3.0199 × 10^−11^	3.0199 × 10^−11^	10/0/0
	100	3.0199 × 10^−11^	3.0199 × 10^−11^	3.0199 × 10^−11^	3.0199 × 10^−11^	3.0199 × 10^−11^	6.5183 × 10^−9^	3.0199 × 10^−11^	3.0199 × 10^−11^	3.0199 × 10^−11^	3.0199 × 10^−11^	10/0/0
	500	3.0199 × 10^−11^	3.0199 × 10^−11^	3.0199 × 10^−11^	3.0199 × 10^−11^	3.0199 × 10^−11^	2.0338 × 10^−9^	3.0199 × 10^−11^	3.0199 × 10^−11^	3.0199 × 10^−11^	3.0199 × 10^−11^	10/0/0
F13	30	3.0029 × 10^−11^	3.0029 × 10^−11^	3.0029 × 10^−11^	3.0029 × 10^−11^	3.0029 × 10^−11^	3.0029 × 10^−11^	3.0029 × 10^−11^	3.0029 × 10^−11^	3.0029 × 10^−11^	3.0029 × 10^−11^	10/0/0
	100	3.0142 × 10^−11^	3.0142 × 10^−11^	3.0142 × 10^−11^	3.0142 × 10^−11^	3.0142 × 10^−11^	3.0142 × 10^−11^	3.0142 × 10^−11^	3.0142 × 10^−11^	3.0142 × 10^−11^	3.0142 × 10^−11^	10/0/0
	500	3.0123 × 10^−11^	3.0123 × 10^−11^	3.0123 × 10^−11^	3.0123 × 10^−11^	3.0123 × 10^−11^	3.0123 × 10^−11^	3.0123 × 10^−11^	3.0123 × 10^−11^	3.0123 × 10^−11^	3.0123 × 10^−11^	10/0/0
F14	2	1.4532 × 10^−1^	1.3853 × 10^−6^	1.8070 × 10^−1^	6.2828 × 10^−6^	2.8790 × 10^−6^	1.7486 × 10^−4^	1.4435 × 10^−10^	5.4485 × 10^−9^	3.8202 × 10^−10^	3.0199 × 10^−11^	5/2/3
F15	4	3.0199 × 10^−11^	3.0199 × 10^−11^	3.0180 × 10^−11^	8.4180 × 10^−1^	5.5546 × 10^−2^	6.3533 × 10^−2^	3.9874 × 10^−4^	6.1452 × 10^−2^	1.6813 × 10^−4^	3.0199 × 10^−11^	5/4/1
F16	2	1.2624 × 10^−11^	1.2624 × 10^−11^	7.2549 × 10^−11^	1.2624 × 10^−11^	1.2624 × 10^−11^	1.3070 × 10^−2^	1.0374 × 10^−4^	1.2624 × 10^−11^	1.2624 × 10^−11^	1.2624 × 10^−11^	7/3/0
F17	2	1.2118 × 10^−12^	1.2118 × 10^−12^	NaN	1.2118 × 10^−12^	1.2118 × 10^−12^	NaN	NaN	1.2118 × 10^−12^	1.2118 × 10^−12^	1.2118 × 10^−12^	7/3/0
F18	2	2.9561 × 10^−11^	2.9561 × 10^−11^	9.1184 × 10^−12^	2.9561 × 10^−11^	2.9561 × 10^−11^	1.6701 × 10^−2^	5.1977 × 10^−7^	2.9561 × 10^−11^	2.9561 × 10^−11^	2.9561 × 10^−11^	7/0/3
F19	3	1.2007 × 10^−11^	1.2007 × 10^−11^	3.6197 × 10^−13^	1.2007 × 10^−11^	1.2007 × 10^−11^	3.7428 × 10^−5^	1.1707 × 10^−9^	1.2007 × 10^−11^	1.2007 × 10^−11^	1.2007 × 10^−11^	7/0/3
F20	6	3.0199 × 10^−11^	1.7769 × 10^−10^	7.2389 × 10^−2^	4.0840 × 10^−5^	5.4941 × 10^−11^	8.0429 × 10^−5^	6.5763 × 10^−1^	9.8329 × 10^−8^	3.0199 × 10^−11^	3.0199 × 10^−11^	7/2/1
F21	4	3.0199 × 10^−11^	1.6225 × 10^−1^	3.7558 × 10^−1^	7.9782 × 10^−2^	4.0840 × 10^−5^	3.4362 × 10^−5^	1.0000 × 10^0^	2.2780 × 10^−5^	9.7555 × 10^−10^	3.0199 × 10^−11^	5/4/1
F22	4	3.0199 × 10^−11^	4.6558 × 10^−7^	1.8361 × 10^−1^	1.1937 × 10^−6^	4.1997 × 10^−10^	2.6947 × 10^−1^	1.0000 × 10^0^	3.3520 × 10^−8^	3.3384 × 10^−11^	3.0199 × 10^−11^	7/3/0
F23	4	3.0199 × 10^−11^	1.0154 × 10^−6^	3.7432 × 10^−1^	7.2208 × 10^−6^	3.0103 × 10^−7^	3.2458 × 10^−1^	7.7028 × 10^−6^	2.8314 × 10^−8^	3.3384 × 10^−11^	3.0199 × 10^−11^	7/2/1

**Table 9:** Results and comparison of CEC 2019 benchmark functions.

F(x)		GA	PSO	ACO	GWO	GJO	SO	TACPSO	AGWO	EGWO	RSA	FRSA
F1	Mean	8.8777 × 10^7^	1.4936 × 10^7^	1.0333 × 10^6^	2.7611 × 10^4^	7.4643 × 10^3^	7.7800 × 10^4^	2.1715 × 10^5^	**1.0000 × 10^0^**	4.8953 × 10^4^	**1.0000 × 10^0^**	**1.0000 × 10^0^**
	Std	9.8039 × 10^7^	3.0138 × 10^7^	8.6424 × 10^5^	8.1437 × 10^4^	3.0692 × 10^4^	1.4879 × 10^5^	2.3490 × 10^5^	**0.0000 × 10^0^**	1.3274 × 10^5^	**0.0000 × 10^0^**	**0.0000 × 10^0^**
F2	Mean	7.7940 × 10^3^	4.1175 × 10^3^	2.6302 × 10^3^	4.7378 × 10^2^	1.6396 × 10^2^	2.8924 × 10^2^	3.3182 × 10^2^	8.2891 × 10^2^	1.6188 × 10^3^	4.9991 × 10^0^	**4.9473 × 10^0^**
	Std	2.5938 × 10^3^	2.4895 × 10^3^	1.7641 × 10^3^	2.2747 × 10^2^	2.7699 × 10^2^	1.7559 × 10^2^	1.3204 × 10^2^	1.7985 × 10^3^	6.1388 × 10^2^	**5.0323 × 10^−3^**	1.0717 × 10^−1^
F3	Mean	1.1095 × 10^1^	8.7904 × 10^0^	5.9218 × 10^0^	**2.9330 × 10^0^**	4.4288 × 10^0^	4.4866 × 10^0^	2.9652 × 10^0^	5.9654 × 10^0^	9.0727 × 10^0^	8.0766 × 10^0^	4.9149 × 10^0^
	Std	9.1758 × 10^−1^	1.2335 × 10^0^	2.1958 × 10^0^	2.0613 × 10^0^	2.5341 × 10^0^	1.9873 × 10^0^	1.8614 × 10^0^	1.1606 × 10^0^	1.9015 × 10^0^	**7.9195 × 10^−1^**	7.9953 × 10^−1^
F4	Mean	3.6379 × 10^1^	4.0010 × 10^1^	2.7100 × 10^1^	1.9449 × 10^1^	3.2685 × 10^1^	2.0590 × 10^1^	**1.8148 × 10^1^**	5.8095 × 10^1^	5.6902 × 10^1^	8.9836 × 10^1^	3.4525 × 10^1^
	Std	1.3237 × 10^1^	7.7174 × 10^0^	1.1349 × 10^1^	1.1020 × 10^1^	1.1314 × 10^1^	**6.0431 × 10^0^**	7.9248 × 10^0^	1.0062 × 10^1^	2.6763 × 10^1^	1.3727 × 10^1^	8.6094 × 10^0^
F5	Mean	6.4731 × 10^0^	3.9550 × 10^0^	1.4494 × 10^0^	2.1800 × 10^0^	3.8695 × 10^0^	1.1470 × 10^0^	**1.1306 × 10^0^**	1.3549 × 10^1^	1.3395 × 10^1^	8.1605 × 10^1^	1.6984 × 10^0^
	Std	5.5146 × 10^0^	3.9669 × 10^0^	2.2462 × 10^−1^	1.1006 × 10^0^	2.6155 × 10^0^	1.5675 × 10^−1^	**7.2699 × 10^−2^**	7.7538 × 10^0^	1.6464 × 10^1^	1.8506 × 10^1^	1.8171 × 10^−1^
F6	Mean	7.8132 × 10^0^	6.6746 × 10^0^	2.9025 × 10^0^	2.7449 × 10^0^	4.5758 × 10^0^	3.8464 × 10^0^	2.5324 × 10^0^	7.6616 × 10^0^	7.7690 × 10^0^	1.0850 × 10^1^	**2.4455 × 10^0^**
	Std	1.6857 × 10^0^	2.3143 × 10^0^	1.3796 × 10^0^	1.2443 × 10^0^	1.1042 × 10^0^	1.2605 × 10^0^	1.2228 × 10^0^	1.1028 × 10^0^	2.1973 × 10^0^	9.5171 × 10^−1^	**7.5682 × 10^−1^**
F7	Mean	1.1598 × 10^3^	1.2966 × 10^3^	7.5342 × 10^2^	8.1406 × 10^2^	1.2074 × 10^3^	**6.9743 × 10^2^**	7.4926 × 10^2^	1.5199 × 10^3^	1.2985 × 10^3^	1.7713 × 10^3^	1.3839 × 10^3^
	Std	3.7858 × 10^2^	3.3549 × 10^2^	4.9219 × 10^2^	3.2861 × 10^2^	4.4397 × 10^2^	2.1649 × 10^2^	3.1405 × 10^2^	2.4732 × 10^2^	3.4208 × 10^2^	**1.8725 × 10^2^**	2.8138 × 10^2^
F8	Mean	5.1737 × 10^0^	4.5708 × 10^0^	3.9766 × 10^0^	3.8578 × 10^0^	4.2642 × 10^0^	3.9505 × 10^0^	**3.8528 × 10^0^**	4.7386 × 10^0^	4.4702 × 10^0^	4.8492 × 10^0^	4.2121 × 10^0^
	Std	2.7203 × 10^−1^	3.3748 × 10^−1^	4.1346 × 10^−1^	4.8374 × 10^−1^	3.3761 × 10^−1^	3.3910 × 10^−1^	3.0223 × 10^−1^	2.7315 × 10^−1^	4.2299 × 10^−1^	**2.4832 × 10^−1^**	2.6721 × 10^−1^
F9	Mean	1.4357 × 10^0^	1.5591 × 10^0^	1.2542 × 10^0^	1.2314 × 10^0^	1.2813 × 10^0^	1.3432 × 10^0^	**1.1940 × 10^0^**	1.5505 × 10^0^	1.3960 × 10^0^	3.2085 × 10^0^	1.2964 × 10^0^
	Std	1.9960 × 10^−1^	3.5904 × 10^−1^	**5.2841 × 10^−2^**	7.6053 × 10^−2^	7.8476 × 10^−2^	8.8253 × 10^−2^	8.5805 × 10^−2^	3.4225 × 10^−1^	1.3667 × 10^−1^	6.4471 × 10^−1^	6.0916 × 10^−2^
F10	Mean	2.1548 × 10^1^	2.1479 × 10^1^	2.1494 × 10^1^	2.1445 × 10^1^	2.1172 × 10^1^	2.1477 × 10^1^	2.0500 × 10^1^	2.1011 × 10^1^	2.1279 × 10^1^	2.1425 × 10^1^	**2.0393 × 10^1^**
	Std	1.1314 × 10^−1^	1.5156 × 10^−1^	1.0565 × 10^−1^	9.7992 × 10^−2^	1.7941 × 10^0^	**8.0458 × 10^−2^**	3.4673 × 10^0^	1.3787 × 10^0^	1.1003 × 10^−1^	1.2053 × 10^−1^	2.2190 × 10^0^
Friedman value		8.4500 × 10^0^	8.0000 × 10^0^	6.3000 × 10^0^	4.7500 × 10^0^	5.7500 × 10^0^	4.4500 × 10^0^	3.8500 × 10^0^	6.5500 × 10^0^	7.9000 × 10^0^	6.4500 × 10^0^	**3.5500 × 10^0^**
Friedman rank		11	10	6	4	5	3	2	8	9	7	**1**

**Table 10:** Statistical analysis results of Wilcoxon rank sum test of CEC 2019 functions.

F(x)	Dim	GA	PSO	ACO	GWO	GJO	SO	TACPSO	AGWO	EGWO	RSA	Total
F1	9	1.2118 × 10^−12^	1.2118 × 10^−12^	1.2118 × 10^−12^	1.2118 × 10^−12^	1.2118 × 10^−12^	1.2118 × 10^−12^	1.2118 × 10^−12^	1.2118 × 10^−12^	NaN	NaN	8/2/0
F2	16	2.5206 × 10^−11^	2.5206 × 10^−11^	2.5206 × 10^−11^	2.5206 × 10^−11^	6.2862 × 10^−8^	2.5206 × 10^−11^	2.5206 × 10^−11^	2.5206 × 10^−11^	9.0983 × 10^−2^	3.0922 × 10^−4^	9/1/0
F3	18	3.0199 × 10^−11^	3.0199 × 10^−11^	3.3386 × 10^−3^	2.1327 × 10^−5^	3.2651 × 10^−2^	2.2823 × 10^−1^	4.7445 × 10^−6^	2.4386 × 10^−9^	5.2640 × 10^−4^	3.6897 × 10^−11^	6/1/3
F4	10	9.7052 × 10^−1^	3.4029 × 10^−1^	2.3985 × 10^−1^	4.1127 × 10^−7^	4.3584 × 10^−2^	3.5201 × 10^−7^	1.5964 × 10^−7^	2.3885 × 10^−4^	3.4971 × 10^−9^	3.0199 × 10^−11^	3/3/4
F5	10	3.0199 × 10^−11^	3.3384 × 10^−11^	7.1988 × 10^−5^	5.2978 × 10^−1^	2.3768 × 10^−7^	3.4742 × 10^−10^	3.0199 × 10^−11^	4.4440 × 10^−7^	3.0199 × 10^−11^	3.0199 × 10^−11^	6/1/3
F6	10	3.0199 × 10^−11^	8.9934 × 10^−11^	3.5545 × 10^−1^	6.9522 × 10^−1^	3.4971 × 10^−9^	2.4327 × 10^−5^	6.6273 × 10^−1^	3.0199 × 10^−11^	3.0199 × 10^−11^	3.0199 × 10^−11^	7/3/0
F7	10	1.0315 × 10^−2^	3.2553 × 10^−1^	5.8587 × 10^−6^	1.0666 × 10^−7^	1.3732 × 10^−1^	8.8910 × 10^−10^	7.7725 × 10^−9^	1.9073 × 10^−1^	7.4827 × 10^−2^	1.2541 × 10^−7^	1/4/5
F8	10	3.3384 × 10^−11^	7.2951 × 10^−4^	1.8916 × 10^−4^	2.8389 × 10^−4^	2.8378 × 10^−1^	6.3772 × 10^−3^	4.4272 × 10^−3^	9.0688 × 10^−3^	8.1200 × 10^−4^	1.3289 × 10^−10^	5/1/4
F9	10	4.2259 × 10^−3^	2.0283 × 10^−7^	6.0971 × 10^−3^	1.8575 × 10^−3^	5.9969 × 10^−1^	4.8413 × 10^−2^	6.2828 × 10^−6^	1.2362 × 10^−3^	1.4110 × 10^−9^	3.0199 × 10^−11^	6/1/3
F10	10	6.2027 × 10^−4^	9.5207 × 10^−4^	1.6813 × 10^−4^	4.4272 × 10^−3^	2.4157 × 10^−2^	2.2360 × 10^−2^	1.0188 × 10^−5^	2.7548 × 10^−3^	1.0547 × 10^−1^	3.3874 × 10^−2^	7/1/2

**Table 11:** Comparison results of pressure vessel design problem.

Algorithms	$x_{1}$	$x_{2}$	$x_{3}$	$x_{4}$	Best Value
GA	1.1943 × 10^0^	5.6359 × 10^−1^	5.6935 × 10^1^	5.4332 × 10^1^	7.4044 × 10^3^
PSO	7.7876 × 10^−1^	3.8637 × 10^−1^	4.0333 × 10^1^	2.0000 × 10^2^	5.8969 × 10^3^
ACO	7.8298 × 10^−1^	3.8703 × 10^−1^	4.0569 × 10^1^	1.9656 × 10^2^	5.8936 × 10^3^
GWO	7.7826 × 10^−1^	3.8541 × 10^−1^	4.0323 × 10^1^	1.9996 × 10^2^	5.8878 × 10^3^
GJO	7.8054 × 10^−1^	3.8666 × 10^−1^	4.0404 × 10^1^	1.9884 × 10^2^	5.8972 × 10^3^
SO	7.7817 × 10^−1^	3.8482 × 10^−1^	4.0320 × 10^1^	2.0000 × 10^2^	5.8858 × 10^3^
TACPSO	7.8287 × 10^−1^	3.8697 × 10^−1^	4.0563 × 10^1^	1.9664 × 10^2^	5.8934 × 10^3^
AGWO	8.0092 × 10^−1^	4.5311 × 10^−1^	4.1339 × 10^1^	1.8843 × 10^2^	6.1686 × 10^3^
EGWO	7.7834 × 10^−1^	3.8642 × 10^−1^	4.0325 × 10^1^	1.9995 × 10^2^	5.8915 × 10^3^
RSA	1.0018 × 10^0^	5.1922 × 10^−1^	4.2327 × 10^1^	1.7775 × 10^2^	7.7528 × 10^3^
**FRSA**	**7.7817 × 10^−1^**	**3.8465 × 10^−1^**	**4.0320 × 10^1^**	**2.0000 × 10^2^**	**5.8854 × 10^3^**

**Table 12:** Statistical analysis of pressure vessel design problem.

Algorithms	Best	Mean	Std	Worst	Time	*p*-Value	
GA	7.4044 × 10^3^	8.8011 × 10^3^	8.6900 × 10^2^	1.1360 × 10^4^	1.7213 × 10^−1^	3.0199 × 10^−11^	+
PSO	5.8969 × 10^3^	6.4337 × 10^3^	6.7244 × 10^2^	7.5156 × 10^3^	1.2070 × 10^−1^	3.7704 × 10^−4^	+
ACO	5.8936 × 10^3^	6.3715 × 10^3^	4.8457 × 10^2^	7.3190 × 10^3^	5.0267 × 10^−1^	1.4733 × 10^−7^	+
GWO	5.8878 × 10^3^	6.0336 × 10^3^	3.2292 × 10^2^	7.2513 × 10^3^	1.3380 × 10^−1^	3.6322 × 10^−1^	=
GJO	5.8972 × 10^3^	6.3251 × 10^3^	5.9094 × 10^2^	7.3194 × 10^3^	2.1300 × 10^−1^	2.2658 × 10^−3^	+
SO	5.8858 × 10^3^	6.2189 × 10^3^	3.3475 × 10^2^	7.1860 × 10^3^	1.4087 × 10^−1^	9.2113 × 10^−5^	+
TACPSO	5.8934 × 10^3^	6.3585 × 10^3^	3.8150 × 10^2^	7.2734 × 10^3^	1.2773 × 10^−1^	1.8500 × 10^−8^	+
AGWO	6.1686 × 10^3^	7.2195 × 10^3^	4.6584 × 10^2^	7.7575 × 10^3^	6.5110 × 10^−1^	3.0199 × 10^−11^	+
EGWO	5.8915 × 10^3^	6.3177 × 10^3^	3.7542 × 10^2^	7.3258 × 10^3^	1.6837 × 10^−1^	3.0939 × 10^−6^	+
RSA	7.7528 × 10^3^	1.2201 × 10^4^	3.2025 × 10^3^	2.0883 × 10^4^	3.1713 × 10^−1^	3.0199 × 10^−11^	+
**FRSA**	**5.8854 × 10^3^**	**5.9418 × 10^3^**	**7.0609 × 10^1^**	**6.1543 × 10^3^**	4.0080 × 10^−1^		

**Table 13:** Comparison of the results for the corrugated bulkhead design problem.

Algorithms	$x_{1}$	$x_{2}$	$x_{3}$	$x_{4}$	Best Value
GA	4.9344 × 10^1^	3.4325 × 10^1^	5.3525 × 10^1^	1.0744 × 10^0^	7.1939 × 10^0^
PSO	5.6734 × 10^1^	3.4160 × 10^1^	5.7676 × 10^1^	1.0502 × 10^0^	6.8516 × 10^0^
ACO	5.7692 × 10^1^	3.4148 × 10^1^	5.7692 × 10^1^	1.0500 × 10^0^	**6.8430 × 10^0^**
GWO	5.7597 × 10^1^	3.4138 × 10^1^	5.7631 × 10^1^	1.0500 × 10^0^	6.8446 × 10^0^
GJO	5.7444 × 10^1^	3.4160 × 10^1^	5.7589 × 10^1^	1.0502 × 10^0^	6.8486 × 10^0^
SO	5.7692 × 10^1^	3.4148 × 10^1^	5.7692 × 10^1^	1.0500 × 10^0^	**6.8430 × 10^0^**
TACPSO	5.7692 × 10^1^	3.4148 × 10^1^	5.7692 × 10^1^	1.0500 × 10^0^	**6.8430 × 10^0^**
AGWO	5.6150 × 10^1^	3.4178 × 10^1^	5.7086 × 10^1^	1.0514 × 10^0^	6.8776 × 10^0^
EGWO	5.7645 × 10^1^	3.4159 × 10^1^	5.7672 × 10^1^	1.0500 × 10^0^	6.8444 × 10^0^
RSA	1.0786 × 10^1^	3.4025 × 10^1^	5.0382 × 10^1^	1.0613 × 10^0^	7.9687 × 10^0^
**FRSA**	5.7692 × 10^1^	3.4148 × 10^1^	5.7692 × 10^1^	1.0500 × 10^0^	**6.8430 × 10^0^**

**Table 14:** Statistical analysis of corrugated bulkhead design problem.

Algorithms	Best	Mean	Std	Worst	Time	*p*-Value	
GA	7.1939 × 10^0^	8.0055 × 10^0^	6.3630 × 10^−1^	1.0132 × 10^1^	1.0340 × 10^−1^	1.4157 × 10^−9^	+
PSO	6.8516 × 10^0^	6.8989 × 10^0^	3.1823 × 10^−2^	6.9810 × 10^0^	4.4200 × 10^−2^	1.4157 × 10^−9^	+
ACO	**6.8430 × 10^0^**	7.4451 × 10^0^	8.3118 × 10^−1^	1.0239 × 10^1^	4.1200 × 10^−1^	2.5585 × 10^−2^	+
GWO	6.8446 × 10^0^	6.8501 × 10^0^	5.4757 × 10^−3^	6.8650 × 10^0^	5.8440 × 10^−2^	1.4157 × 10^−9^	+
GJO	6.8486 × 10^0^	7.2569 × 10^0^	6.4078 × 10^−1^	8.2682 × 10^0^	1.3556 × 10^−1^	1.4157 × 10^−9^	+
SO	**6.8430 × 10^0^**	6.8432 × 10^0^	7.1300 × 10^−4^	6.8460 × 10^0^	6.1040 × 10^−2^	1.2780 × 10^−3^	+
TACPSO	**6.8430 × 10^0^**	6.9001 × 10^0^	2.8554 × 10^−1^	8.2707 × 10^0^	4.8960 × 10^−2^	2.1634 × 10^−8^	-
AGWO	6.8776 × 10^0^	7.0434 × 10^0^	2.5644 × 10^−1^	8.1805 × 10^0^	4.8984 × 10^−1^	1.4157 × 10^−9^	+
EGWO	6.8444 × 10^0^	6.9353 × 10^0^	2.8175 × 10^−1^	8.1632 × 10^0^	8.8400 × 10^−2^	1.4157 × 10^−9^	+
RSA	7.9687 × 10^0^	9.1028 × 10^0^	8.3088 × 10^−1^	1.0716 × 10^1^	2.1428 × 10^−1^	1.4157 × 10^−9^	+
**FRSA**	**6.8430 × 10^0^**	**6.8430 × 10^0^**	**1.0000 × 10^−7^**	**6.8430 × 10^0^**	1.8084 × 10^−1^		

**Table 15:** Comparison of the results for the welded beam design problem.

Algorithms	$x_{1}$	$x_{2}$	$x_{3}$	$x_{4}$	Best Value
GA	1.7200 × 10^−1^	4.7314 × 10^0^	8.7256 × 10^0^	2.2693 × 10^−1^	1.9390 × 10^0^
PSO	2.0560 × 10^−1^	3.4728 × 10^0^	9.0405 × 10^0^	2.0588 × 10^−1^	1.7268 × 10^0^
ACO	2.0632 × 10^−1^	3.4629 × 10^0^	9.0235 × 10^0^	2.0633 × 10^−1^	1.7270 × 10^0^
GWO	2.0547 × 10^−1^	3.4781 × 10^0^	9.0365 × 10^0^	2.0574 × 10^−1^	1.7256 × 10^0^
GJO	2.0557 × 10^−1^	3.4733 × 10^0^	9.0418 × 10^0^	2.0573 × 10^−1^	1.7259 × 10^0^
SO	2.0573 × 10^−1^	3.4705 × 10^0^	9.0368 × 10^0^	2.0573 × 10^−1^	**1.7249 × 10^0^**
TACPSO	2.0573 × 10^−1^	3.4705 × 10^0^	9.0366 × 10^0^	2.0573 × 10^−1^	**1.7249 × 10^0^**
AGWO	2.0261 × 10^−1^	3.5867 × 10^0^	9.0420 × 10^0^	2.0573 × 10^−1^	1.7366 × 10^0^
EGWO	2.0538 × 10^−1^	3.4793 × 10^0^	9.0370 × 10^0^	2.0573 × 10^−1^	1.7256 × 10^0^
RSA	2.0413 × 10^−1^	3.3786 × 10^0^	1.0000 × 10^1^	2.0723 × 10^−1^	1.8881 × 10^0^
**FRSA**	2.0573 × 10^−1^	3.4705 × 10^0^	9.0366 × 10^0^	2.0573 × 10^−1^	**1.7249 × 10^0^**

**Table 16:** Statistical analysis of welded beam design problem.

Algorithms	Best	Mean	Std	Worst	Time	*p*-Value	
GA	1.9390 × 10^0^	3.2100 × 10^0^	9.8809 × 10^−1^	5.6391 × 10^0^	2.0927 × 10^−1^	3.0199 × 10^−11^	+
PSO	1.7268 × 10^0^	1.8233 × 10^0^	2.1321 × 10^−1^	2.4983 × 10^0^	1.5083 × 10^−1^	3.0199 × 10^−11^	+
ACO	1.7270 × 10^0^	2.1779 × 10^0^	4.3684 × 10^−1^	3.7688 × 10^0^	5.3960 × 10^−1^	3.0199 × 10^−11^	+
GWO	1.7256 × 10^0^	1.7281 × 10^0^	2.9589 × 10^−3^	1.7368 × 10^0^	1.6927 × 10^−1^	3.0199 × 10^−11^	+
GJO	1.7259 × 10^0^	1.7303 × 10^0^	4.2440 × 10^−3^	1.7429 × 10^0^	2.4570 × 10^−1^	3.0199 × 10^−11^	+
SO	**1.7249 × 10^0^**	1.7278 × 10^0^	6.9340 × 10^−3^	1.7533 × 10^0^	1.7103 × 10^−1^	1.8608 × 10^−6^	+
TACPSO	**1.7249 × 10^0^**	1.7504 × 10^0^	5.3535 × 10^−2^	1.9215 × 10^0^	1.5860 × 10^−1^	4.2039 × 10^−1^	=
AGWO	1.7366 × 10^0^	1.7725 × 10^0^	1.8314 × 10^−2^	1.8307 × 10^0^	6.9630 × 10^−1^	3.0199 × 10^−11^	+
EGWO	1.7256 × 10^0^	1.7305 × 10^0^	4.7509 × 10^−3^	1.7462 × 10^0^	2.0027 × 10^−1^	3.0199 × 10^−11^	+
RSA	1.8881 × 10^0^	2.1518 × 10^0^	1.6396 × 10^−1^	2.6872 × 10^0^	3.5377 × 10^−1^	3.0199 × 10^−11^	+
**FRSA**	**1.7249 × 10^0^**	**1.7249 × 10^0^**	**5.6900 × 10^−5^**	**1.7252 × 10^0^**	4.8907 × 10^−1^		

## Data Availability

Not applicable.
